# 3-Nitroindoles Serving as *N*-Centered Nucleophiles for Aza-1,6-Michael Addition to *para*-Quinone Methides

**DOI:** 10.3390/molecules28145529

**Published:** 2023-07-20

**Authors:** Jian-Qiang Zhao, Wen-Jie Wang, Shun Zhou, Qi-Lin Xiao, Xi-Sha Xue, Yan-Ping Zhang, Yong You, Zhen-Hua Wang, Wei-Cheng Yuan

**Affiliations:** 1 Innovation Research Center of Chiral Drugs, Institute for Advanced Study, Chengdu University, Chengdu 610106, China; 2 National Engineering Research Center of Chiral Drugs, Chengdu Institute of Organic Chemistry, Chinese Academy of Sciences, Chengdu 610041, China

**Keywords:** 3-nitroindoles, *N*-alkylation, *para*-quinone methides, *aza*-1,6-Michael addition, indole derivatives

## Abstract

An unprecedented *N*-alkylation of 3-nitroindoles with *para*-quinone methides was developed for the first time. Using potassium carbonate as the base, a wide range of structurally diverse *N*-diarylmethylindole derivatives were obtained with moderated to good yields via the protection group migration/*aza*-1,6-Michael addition sequences. The reaction process was also demonstrated by control experiments. Different from the previous advances where 3-nitrodoles served as electrophiles trapping by various nucleophiles, the reaction herein is featured that 3-nitrodoles is defined with latent *N*-centered nucleophiles to react with *ortho*-hydrophenyl *p*-QMs for construction of various *N*-diarylmethylindoles.

## 1. Introduction

**Scheme 1:**
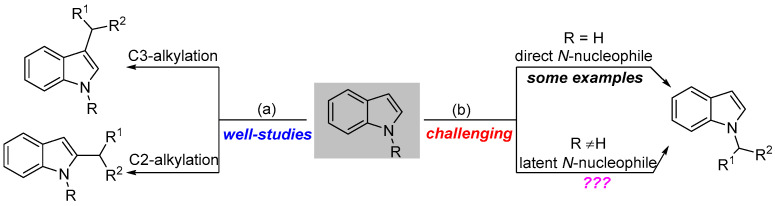
The strategies of direct alkylation of indoles. (**a**) C2 and C3-alkylation of indoles; (**b**) *N*-alkylation of indoles.

Indole-based motifs are privileged structures for construction of various valuable complex heteroaromatic units, which widely exist in numerous biologically active natural products and pharmaceutically relevant compounds with potential pharmacological activities, such as against cancer, HIV, inflammation, tuberculosis, hypertension, diabetes, and against microbial, viral, and fungal infections [1,2,3,4,5,6]. As a result, enormous efforts have been devoted to exploring versatile techniques for the efficient synthesis of structurally diverse indole derivatives, and thus the indole-based chemistry has become a hotspot in organic synthesis [7,8,9,10,11,12]. In conventional indole alkylation reactions, the most common synthetic modifications occurred at the C2 and C3-positions of indoles due to the innate nucleophilic nature (Scheme 1a) [13,14]. In contrast, the *N*-alkylation of indoles is challenging, and only a few reports have been disclosed to directly fabricate such compounds (Scheme 1b) [15,16]. Among the established *N*-alkylation of indoles, the studies mainly focused on modification of *N*-H indole derivatives by taking advantage of the nucleophilicity of the nitrogen atom [17,18,19,20,21,22,23]. However, the weak nucleophilicity of the nitrogen atom in indoles commonly resulted in the C2 or C3-positions alkylated by-products. In order to increase the *N*-centered nucleophilicity, an alternative method is the introduction of a protecting group at the *N*1-position of indoles made it latent nucleophiles [24,25,26,27], which are themselves not nucleophilic but can produce a strong nucleophile in situ via deprotection. To the best of our knowledge, it was only in 2019 that the Vilotijevic group reported that *N*-silyl indoles were employed as latent *N*-centered nucleophiles in the substitution of allylic fluorides for *N*-allyl indoles [28]. Therefore, the exploration of *N*-protected indoles as latent *N*-centered nucleophiles in *N*-alkylation reaction is huge challenges.

**Scheme 2:**
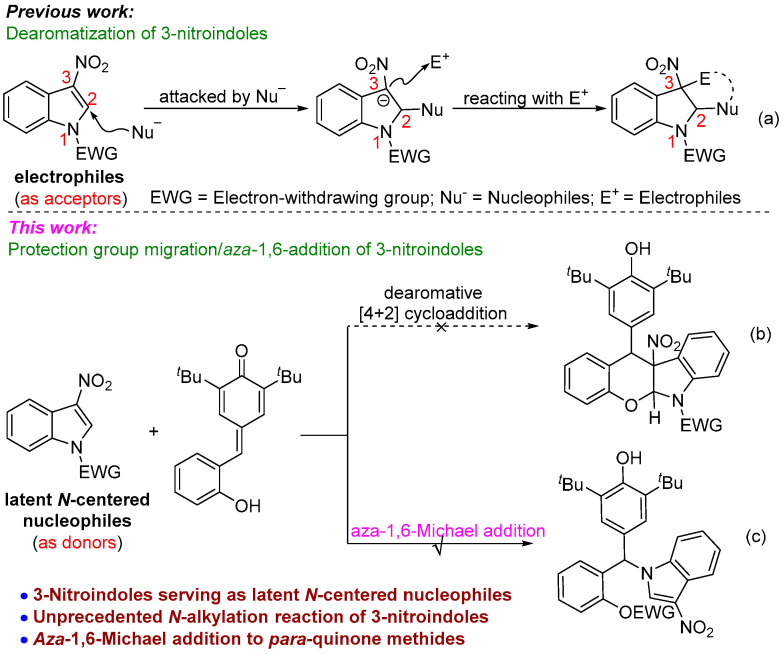
The profile of dearomatization of 3-nitroindoles and this work on unprecedented *N*-alkylation of 3-nitroindoles. (**a**) The reaction feature of 3-nitroindoles; (**b**) The expected dearomative [4 + 2] cycloaddition of 3-nitroindoles; (**c**) The unanticipated *N*-alkylation of 3-nitroindoles in this work.

In recent years, there have an increasing number of reports on 3-nitroindoles as electrophiles in the reaction with various nucleophiles for the construction of diverse indolines via dearomative process [29,30,31,32,33]. Among these reactions, 3-nitroindoles are characterized by their readiness to be attacked by nucleophiles at the C2-position and sequentially trapped by electrophiles with the C3-position for the synthesis of indoline-containing polycyclic compounds (Scheme 2a). On the other hand, we have noticed that in the field of *para*-quinone methides (*p*-QMs) chemistry [34,35,36,37], *ortho*-hydrophenyl *p*-QMs have been used as donors to trigger some cycloaddition reactions with electron-deficient 2π-components, providing an access to chromans with structural diversity [38,39,40,41,42,43,44]. Along this line, as well as our continuing efforts on the dearomatization of nitroheteroarenes [45,46,47,48], we conceived that the dearomative [4 + 2] cycloaddition of electron-deficient 3-nitroindoles and *ortho*-hydrophenyl *p*-QMs might occur via the tandem *oxy*-Michael addition/1,6-addition under alkaline conditions (Scheme 2b) [49]. To our surprise, the reaction between 3-nitroindoles and *ortho*-hydrophenyl *p*-QMs did undergo smoothly but providing unanticipated *N*-alkylation products via protection group migration/*aza*-1,6-Michael addition pathway instead of the dearomative [4 + 2] cyclo-adducts (Scheme 2c). In this manuscript, the *N*-protected 3-nitroindoles served as latent *N*-centered nucleophiles to couple with *ortho*-hydrophenyl *p*-QMs and the protecting group was transferred from the *N*-center of indoles to the *O*-center of *ortho*-hydrophenyl *p*-QMs, leading to the *N*-diarylmethylindoles with good yields. Obviously, different from the previous advances where 3-nitroindoles serving as electrophiles were attacked by various nucleophiles, the reaction herein is featured that 3-nitrodoles is defined with latent *N*-centered nucleophiles to react with *ortho*-hydrophenyl *p*-QMs. Herein, we wish to reported the initial finds toward this protection group migration/*aza*-1,6-Michael addition sequences.

## 2. Results and Discussion

### 2.1. Optimization Studies

We started our research with the selection of *N*-Ts 3-nitroindole **1a** and *ortho*-hydroxyphenyl-substituted *para*-quinone methide **2a** as the model substrates for optimizing the reaction conditions (Table 1). Using 1,4-diazabicyclo[2.2.2]octane (DABCO) as the base, the desired *N*-alkylated product **3a** was obtained in 44% yield in toluene at 50 °C for 7 days (entry 1). However, when DABCO was replaced with stronger organic base 1,8-diazabicyclo[5.4.0]undec-7-ene (DBU), a reduced yield was observed (entry 2). We then tested different inorganic bases such as Na_2_CO_3_ and K_2_CO_3_ (entries 3 and 4), and it was found that K_2_CO_3_ was the best candidate, giving the product **3a** with a yield of 60% (entry 4). Afterward, various solvents including CH_2_Cl_2_, THF, EtOAc, CH_3_CN and MeOH were examined, and it was found that CH_3_CN was the best choice to give the product **3a** in 67% yield (entry 8 vs. entries 5–7 and 9). By cooling the reaction temperature to room temperature (rt), the yield of **3a** could be increased to 71% (entry 10). A slightly lower yield was obtained when the amount of K_2_CO_3_ was reduced to 1.0 equivalent (entry 11). Though a series of detailed investigations, the reaction conditions were eventually optimized as follows: 1.0 mmol of **1a** and 1.0 mmol of **2a**, 2.0 equiv. of K_2_CO_3_ as base in CH_3_CN as solvent at room temperature.

### 2.2. Substrate Scope Studies

**Scheme 3:**
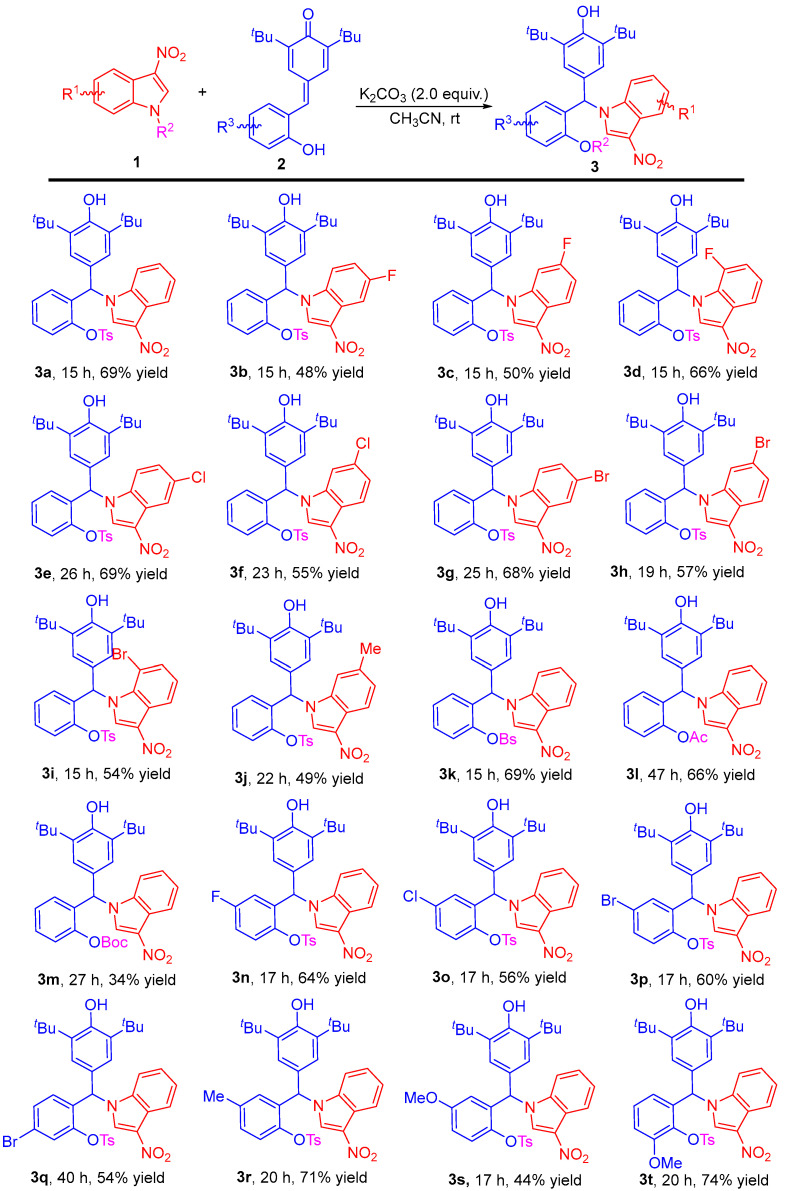
**Substrate scope of *ortho*-hydroxy *p*-QMs and 3-nitroindoles.** Reaction conditions: the reactions were carried out with **1** (0.1 mmol), **2** (0.1 mmol) and K_2_CO_3_ (2.0 equiv) in 1.0 mL of CH_3_CN at room temperature. The yield refers to the isolated yield.

With the optimal reaction conditions in hand, we next surveyed the scope and generality for the *N*-alkylation of 3-nitroindoles with *para*-quinone methides. As shown in Scheme 3, by installing a fluorine atom into the aromatic ring at the C5-, C6- or C7-position of *N*-Ts-3-nitroindoles, these reactions proceeded well to provide the corresponding products **3b**–**d** in moderate yields. Moreover, 3-nitroindoles bearing different electron-withdrawing group, such as Cl- and Br-, regardless of their position on the aromatic ring, could react smoothly with **2a** to deliver products **3e**–**i** in satisfactory results. Nevertheless, the 3-nitroindole bearing a methyl group on the aromatic ring was also viable under the standard conditions, as demonstrated by the formation of product **3j** in 49% yield. Changing the *N*1-proceting group of 3-nitroindole from -Ts to -Bs, had little effect on the reactivity, which could react smoothly with *ortho*-hydroxyphenyl-substituted *para*-quinone methide **2a,** providing the corresponding product **3k** in 69% yield. In addition, the developed catalytic system was also compatible with the *N*-Ac and *N*-alkoxycarbonylated protected 3-nitroindoles, generating the desired products **3l** and **3m** in acceptable yields via tandem protection group migration/*aza*-1,6-Michael addition sequences. On the other hand, various *ortho*-hydroxy *p*-QMs with either electron-withdrawing or -donating groups in the phenyl ring irrespective of their position were well tolerated to provide the expected products **3n**–**t** in moderate to good yields.

### 2.3. Scale-Up Experiment

**Scheme 4:**
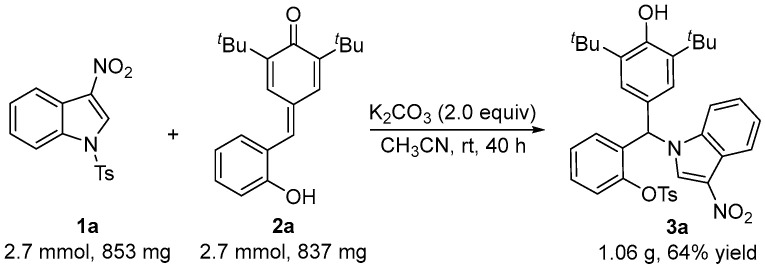
Scale-up experiment.

To demonstrate the synthetic potential of this unprecedented *N*-alkylation of 3-nitroindoles and *para*-quinone methides, a scale-up experiment was performed between **1a** and **2a**, which is 27 times larger than the scale of the model reaction in Scheme 3. As shown in Scheme 4, the gram-scale reaction proceeded well under the standard conditions and afforded the desired product **3a** in 64% yield, suggesting that the developed protocol has good scalability in organic synthesis.

### 2.4. Control Experiments

**Scheme 5:**
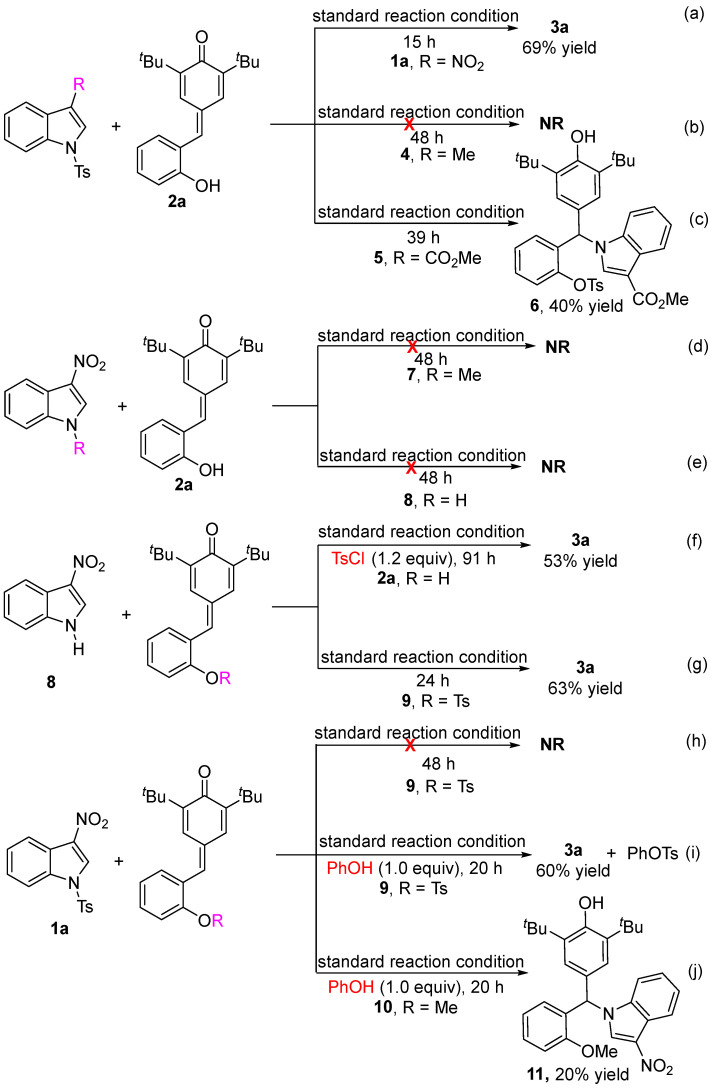
Control experiments.

In order to clarify the possible reaction mechanism, some control experiments were carried out (Scheme 5). The reaction of **1a** and **2a** provided the desired *N*-alkylated product **3a** in 69% yield under the standard reaction conditions (Scheme 5a). Changing the nitro group of **1a** to methyl resulted in the substrate **4** being formed, which failed to react with **2a** (Scheme 5b). When the *N*-Ts indole-3-carboxylate **5** was reacted with **2a**, the reaction gave the corresponding product **6** in 40% yield (Scheme 5c). These experimental results show that the installation of an electron-withdrawing group at the C3-posion of indole is crucial for this *aza*-1,6-Michael addition. Furthermore, the effect of the *N*1-protecting group of 3-nitroindole on the reactivity was also investigated (Scheme 5d,e). With the electron-donating group *N*-Me 3-nitroindole **7** or *N*-H 3-nitroindole **8** as the substrate, no desired *N*-alkylated product was detected (Scheme 5d,e). Comparing the results with Scheme 5a, it can be concluded that the *N*1-electron-withdrawing group of indoles plays an important role in assisting migration of *N*-electron-withdrawing group of 3-nitroindoles to *O*-center of *ortho*-hydrophenyl *p*-QMs and forming the *N*-centered nucleophiles. In addition, it was found that the one-pot reaction of *N*-H 3-nitroindole **8**, **2a** and TsCl could give product **3a** in 53% yield (Scheme 5f), and the reaction of *N*-H 3-nitroindole **8** and *ortho*-oTs *p*-QM **9** could also afford **3a** in 63% yield (Scheme 5g). From these two reactions, it can be confirmed that the sulfonylation of *ortho*-hydroxy *p*-QMs could enhance the electrophilicity and facilitate subsequent *aza*-1,6-Michael addition. Moreover, the reaction of *N*-Ts-3-nitroindole **1a** and *ortho*-OTs *p*-QM **9** could not happen under the standard reaction conditions (Scheme 5h). However, by adding 1.0 equivalent PhOH into the reaction system, the reaction was able to give **3a** in 60% yield, together with the formation of PhOTs (Scheme 5i). Meanwhile, the three-component reaction of **1a**, *ortho*-OMe *p*-QM **10** and PhOH also proceeded to give product **11** and PhOTs (Scheme 5j). These control experiments show that the *N*-EWG of **1a** is first transferred to *O*-EWG of **2a** to form **9** under alkaline condition, and then the *aza*-1,6-Michel addition to *para*-quinone methides takes place to give the *N*-alkylated products.

### 2.5. Plausible Reaction Mechanism

**Scheme 6:**
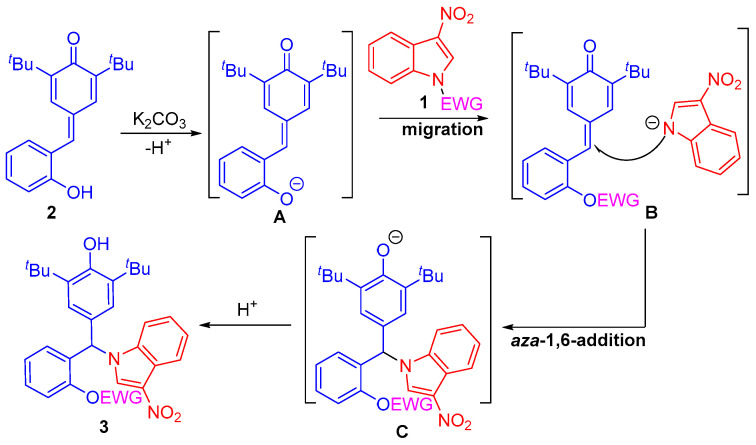
Plausible reaction mechanism.

Based on our experimental results and the above control experiments, a plausible reaction mechanism was proposed for this base-mediated *N*-alkylation of 3-nitroindoles with *para*-quinone methides. As shown in Scheme 6, the initially K_2_CO_3_-promoted deprotonation of *ortho*-hydroxy phenyl *p*-QMs **2** affords intermediate **A**. Then the protecting group was transferred from the *N*-center of indoles to the *O*-center of *ortho*-hydrophenyl *p*-QMs to give *ortho*-OEWG phenyl *p*-QMs and the 3-nitroindole anion intermediates **B**, which undergoes an *aza*-1,6-Michael addition to give the intermediate **C**. Finally, the protonation of intermediate **C** gives rise to the formation of the *N*-alkylated products **3**.

### 2.6. X-ray Crystallographic Structures

**Figure 1:**
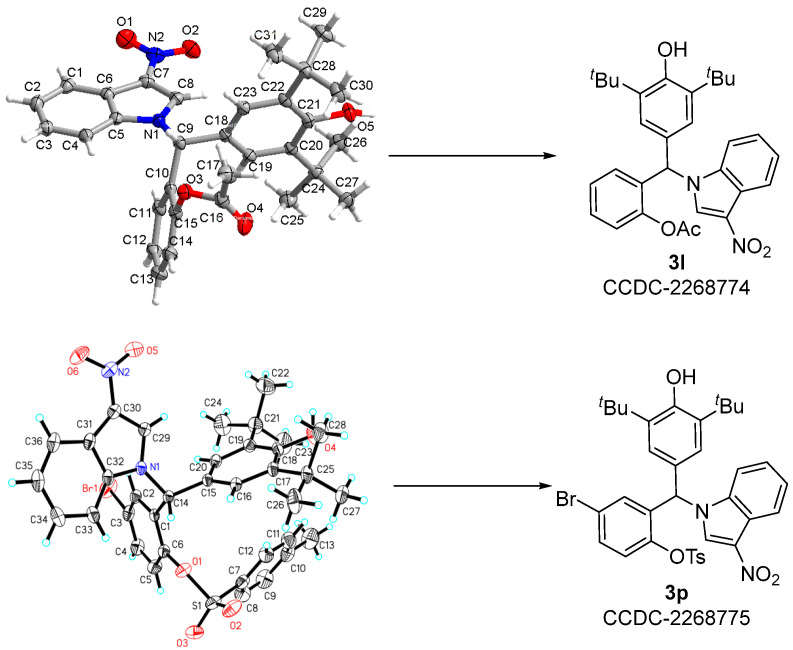
X-ray crystallographic structures of **3l** and **3p**.

All the *N*-alkylation products obtained from the reaction of 3-nitroindoles with *ortho*-hydrophenyl *p*-QMs were unambiguously characterized by nuclear magnetic resonance spectroscopy and high resolution mass spectroscopy. Nevertheless, the structures of products **3l** and **3p** were confirmed by X-ray crystallographic study of the single crystals, which could be readily prepared from the mixture solvents of dichloromethane/EtOH (*V*: *V* = 1/10) at room temperature by slow evaporation of solvents (Figure 1). CCDC-2268774 (**3l**) and CCDC-2268775 (**3p**) contain the supplementary crystallographic data for this paper, which can be obtained free of charge from The Cambridge Crystallographic Data Centre.

## 3. Materials and Methods

### 3.1. General Information

Reagents were purchased from commercial sources and were used as received unless mentioned otherwise. Reactions were monitored by thin layer chromatography (TLC). ^1^H NMR and ^13^C NMR spectra were recorded in CDCl_3_ and DMSO-*d*_6_. ^1^H NMR chemical shifts are reported in ppm relative to tetramethylsilane (TMS) with the solvent resonance employed as the internal standard (CDCl_3_ at 7.26 ppm, DMSO-*d*_6_ at 2.50 ppm). Data are reported as follows: chemical shift, multiplicity (s = singlet, br s = broad singlet, d = doublet, t = triplet, q = quartet, m = multiplet), coupling constants (Hz), and integration. ^13^C NMR chemical shifts are reported in ppm from tetramethylsilane (TMS) with the solvent resonance as the internal standard (CDCl_3_ at 77.20 ppm, DMSO-*d*_6_ at 39.52 ppm). Melting points products were recorded on a Büchi Melting Point B-545. The HRMS were recorded by The HRMS were recorded by Agilent 6545 LC/Q-TOF mass spectrometer.

### 3.2. General Experimental Procedure for the N-Alkylation of 3-Nitroindoles with para-Quinone Methides for the Synthesis of N-Diarylmethylindole Derivatives 3 (Scheme 3)

In a reaction tube equipped with a magnetic stirring bar, the 3-nitroindoles **1** (0.1 mmol, 1 equiv), *ortho-hydroxyphenyl-substituted para-quinone methide* **2** (0.1 mmol, 1.0 equiv), K_2_CO_3_ (0.2 mmol, 2.0 equiv) and acetonitrile (1.0 mL) were added. Then, the mixture was stirred at room temperature. After completion, the mixture was concentrated and purified by flash chromatography on silica gel to give the corresponding products **3**.

**3a**, white solid, 41.8 mg, 69% yield; m.p. 199.5–200.8 °C; ^1^H NMR (400 MHz, Chloroform-d) δ 8.34–8.27 (m, 1 H), 7.70 (s, 1 H), 7.69–7.64 (m, 2 H), 7.43–7.26 (m, 4 H), 7.26–7.17 (m, 4 H), 7.07 (s, 1H), 6.80 (s, 2 H), 6.71 (dd, J = 7.8, 1.7 Hz, 1 H), 5.33 (s, 1 H), 2.42 (s, 3H), 1.33 (s, 18H). ^13^C NMR (101 MHz, Chloroform-*d*) δ 154.3, 147.2, 145.9, 136.7, 135.6, 132.7, 132.0, 130.0, 129.9, 129.9, 128.8, 128.5, 128.1, 127.4, 126.2, 125.4, 124.7, 124.5, 122.4, 121.4, 120.7, 112.2, 60.0, 34.4, 30.1, 21.7. HRMS (ESI-TOF) calcd. for C_36_H_38_N_2_O_6_SNa [M + Na]^+^ 649.2343; found: 649.2357.

**3b**, white solid, 30.9 mg, 48% yield; m.p. 194.4–195.1 °C; ^1^H NMR (400 MHz, Chloroform-*d*) δ 7.96 (dd, *J* = 9.1, 2.5 Hz, 1H), 7.76–7.62 (m, 3H), 7.38–7.15 (m, 6H), 7.10–6.94 (m, 2H), 6.81 (s, 2H), 6.69 (dd, *J* = 7.8, 1.7 Hz, 1H), 5.35 (s, 1H), 2.44 (s, 3H), 1.33 (s, 18H). ^13^C NMR (101 MHz, Chloroform-*d*) δ 160.6 (d, *J* = 241.4 Hz), 154.4, 147.2, 146.0, 136.8, 132.6, 132.0, 131.9, 131.0, 130.0, 130.0, 128.4, 128.1, 127.5, 125.9, 125.3, 122.5, 122.2, 122.1, 113.6, 113.5 (d, *J* = 6.8 Hz), 113.2, 106.4 (d, *J* = 26.4 Hz), 60.4, 34.4, 30.1, 21.7. HRMS (ESI-TOF) calcd. for C_36_H_37_FN_2_O_6_SNa [M + Na]^+^ 667.2249; found: 667.2261.

**3c**, white solid, 32.2 mg, 50% yield; m.p. 190.1–191.4 °C; ^1^H NMR (400 MHz, Chloroform-*d*) δ 8.24 (dd, *J* = 8.8, 5.3 Hz, 1H), 7.73–7.66 (m, 3H), 7.43–7.10 (m, 6H), 7.03 (dd, *J* = 9.3, 2.3 Hz, 1H), 6.94 (s, 1H), 6.80 (s, 2H), 6.69 (dd, *J* = 7.7, 1.7 Hz, 1H), 5.35 (s, 1H), 2.44 (s, 3H), 1.34 (s, 18H). ^13^C NMR (101 MHz, Chloroform-*d*) δ 160.7 (d, *J* = 243.2 Hz), 154.4, 147.3, 146.0, 136.8, 135.7, 135.3, 132.6, 131.7, 130.3 (d, *J* = 2.7 Hz), 130.0, 128.8, 128.4, 128.2, 127.5, 125.8, 125.3, 122.6, 122.0 (d, *J* = 9.8 Hz), 117.8, 113.2 (d, *J* = 24.4 Hz), 99.0 (d, *J* = 27.1 Hz), 60.3, 34.4, 30.1, 21.7. HRMS (ESI-TOF) calcd. for C_36_H_37_FN_2_O_6_SNa [M + Na]^+^ 667.2249; found: 667.2257.

**3d**, white solid, 42.5 mg, 69% yield; m.p. 192.7–193.7 °C; ^1^H NMR (400 MHz, Chloroform-*d*) δ 8.06 (dd, *J* = 8.1, 1.0 Hz, 1H), 7.65 (s, 1H), 7.58–7.51 (m, 2H), 7.49 (dd, *J* = 8.2, 1.3 Hz, 1H), 7.39 (td, *J* = 8.3, 7.9, 1.7 Hz, 1H), 7.28 (s, 1H), 7.28 (td, *J* = 8.1, 4.5 Hz, 1 H), 7.22 (td, *J* = 7.6, 1.3 Hz, 1H), 7.18–7.11 (m, 2H), 7.00–6.91 (m, 1H), 6.74 (dd, *J* = 7.8, 1.7 Hz, 1H), 6.72 (s, 2H), 5.31 (s, 1H), 2.37 (s, 3H), 1.32 (s, 18H). ^13^C NMR (101 MHz, Chloroform-*d*) δ 154.2, 146.7 (d, *J* = 225.5 Hz), 136.5, 132.8, 131.2, 130.6, 130.2, 129.8, 129.3, 128.9, 127.9, 126.9, 125.0, 124.9, 124.6, 123.8, 121.7, 116.6, 116.6, 110.7, 110.6, 61.7 (d, *J* = 7.0 Hz), 34.3, 30.1, 21.7. HRMS (ESI-TOF) calcd. for C_36_H_37_FN_2_O_6_SNa [M + Na]^+^ 667.2249; found: 667.2258.

**3e**, white solid, 45.5 mg, 69% yield; m.p. 174.9–175.4 °C; ^1^H NMR (400 MHz, Chloroform-*d*) δ 8.29 (d, *J* = 2.0 Hz, 1H), 7.72–7.64 (m, 3H), 7.38–7.15 (m, 8H), 7.04 (s, 1H), 6.80 (s, 2H), 6.68 (dd, *J* = 7.8, 1.7 Hz, 1H), 5.36 (s, 1H), 2.44 (s, 3H), 1.33 (s, 18H). ^13^C NMR (101 MHz, Chloroform-*d*) δ 154.5, 147.1, 146.0, 136.8, 133.9, 132.6, 131.8, 130.8, 130.7, 130.1, 130.0, 128.4, 128.2, 128.1, 127.5, 125.8, 125.3, 125.3, 122.5, 122.3, 120.3, 113.4, 60.4, 34.4, 30.1, 21.8. HRMS (ESI-TOF) calcd. for C_36_H_37_ClN_2_O_6_SNa [M + Na]^+^ 683.1953; found: 683.1972.

**3f**, white solid, 436.4 mg, 55% yield; m.p. 234.8–236.0 °C; ^1^H NMR (400 MHz, Chloroform-*d*) δ 8.21 (d, *J* = 8.6 Hz, 1H), 7.75–7.65 (m, 3H), 7.44–7.13 (m, 8H), 6.97 (s, 1H), 6.78 (s, 2H), 6.69 (dd, *J* = 7.8, 1.7 Hz, 1H), 5.36 (s, 1H), 2.44 (s, 3H), 1.34 (s, 18H). ^13^C NMR (101 MHz, Chloroform-*d*) δ 154.5, 147.2, 146.0, 136.8, 135.9, 132.6, 131.6, 130.8, 130.4, 130.2, 130.0, 128.8, 128.5, 128.2, 127.5, 125.8, 125.2, 125.2, 122.7, 121.6, 119.8, 112.2, 60.2, 34.4, 30.1, 21.8. HRMS (ESI-TOF) calcd. for C_36_H_37_ClN_2_O_6_SNa [M + Na]^+^ 683.1953; found: 683.1965.

**3g**, white solid, 47.9 mg, 68% yield; m.p. 238.4–239.7 °C; ^1^H NMR (400 MHz, Chloroform-*d*) δ 8.46 (d, *J* = 1.9 Hz, 1H), 7.67 (d, *J* = 9.0 Hz, 3H), 7.42–7.30 (m, 2H), 7.30–7.13 (m, 6H), 7.04 (s, 1H), 6.80 (s, 2H), 6.68 (dd, *J* = 7.7, 1.6 Hz, 1H), 5.36 (s, 1H), 2.44 (s, 3H), 1.33 (s, 18H). ^13^C NMR (101 MHz, Chloroform-*d*) δ 154.5, 147.1, 146.0, 136.8, 134.2, 132.6, 131.7, 130.6, 130.1, 130.0, 128.4, 128.1, 128.0, 127.9, 127.5, 125.8, 125.3, 123.4, 122.7, 122.5, 118.4, 113.7, 60.4, 34.4, 30.1, 21.8. HRMS (ESI-TOF) calcd. for C_36_H_37_BrN_2_O_6_SNa [M + Na]^+^ 729.1434; found: 729.1445.

**3h**, white solid, 40.1 mg, 57% yield; m.p. 202.6–203.9 °C; ^1^H NMR (400 MHz, Chloroform-*d*) δ 8.16 (d, *J* = 8.6 Hz, 1H), 7.74–7.65 (m, 3H), 7.58 (d, *J* = 1.6 Hz, 1H), 7.50 (dd, *J* = 8.6, 1.6 Hz, 1H), 7.35 (td, *J* = 7.8, 1.7 Hz, 1H), 7.28 (d, *J* = 8.2 Hz, 2H), 7.26–7.16 (m, 2H), 6.98 (s, 1H), 6.78 (s, 2H), 6.70 (dd, *J* = 7.8, 1.7 Hz, 1H), 5.36 (s, 1H), 2.44 (s, 3H), 1.34 (s, 18H). ^13^C NMR (101 MHz, Chloroform-*d*) δ 154.5, 147.2, 146.0, 136.8, 136.2, 132.6, 131.6, 130.3, 130.2, 130.0, 128.8, 128.5, 128.2, 127.8, 127.5, 125.8, 125.2, 122.7, 122.0, 120.1, 118.4, 115.2, 60.2, 34.4, 30.1, 21.8. HRMS (ESI-TOF) calcd. for C_36_H_37_BrN_2_O_6_SNa [M + Na]^+^ 729.1434; found: 729.1442.

**3i**, white solid, 38.0 mg, 54% yield; m.p. 230.2–231.1 °C; ^1^H NMR (400 MHz, Chloroform-*d*) δ 8.03 (d, *J* = 8.1 Hz, 2H), 7.75–7.68 (m, 2H), 7.43–7.32 (m, 3H), 7.17–7.08 (m, 3H), 7.08–6.92 (m, 3H), 6.87 (td, *J* = 7.4, 1.3 Hz, 1H), 5.22 (s, 1H), 4.97 (s, 1H), 2.46 (s, 3H), 1.39 (s, 18H). ^13^C NMR (101 MHz, Chloroform-*d*) δ 153.9, 151.7, 143.9, 142.5, 138.7, 137.3, 136.2, 131.7, 129.3, 129.2, 127.5, 127.2, 126.7, 125.9, 125.4, 125.3, 124.8, 124.1, 117.9, 110.2, 96.6, 92.6, 51.6, 34.4, 30.2, 21.7. HRMS (ESI-TOF) calcd. for C_36_H_37_BrN_2_O_6_SNa [M + Na]^+^ 729.1434; found: 729.1439.

**3j**, white solid, 31.3 mg, 49% yield; m.p. 193.8–194.9 °C; ^1^H NMR (400 MHz, Chloroform-*d*) δ 8.16 (d, *J* = 8.2 Hz, 1H), 7.69–7.62 (m, 3H), 7.37–7.29 (m, 1H), 7.29–7.15 (m, 6H), 7.04 (s, 1H), 6.79 (s, 2H), 6.72 (dd, *J* = 7.8, 1.7 Hz, 1H), 5.32 (s, 1H), 2.44 (s, 3H), 2.42 (s, 3H), 1.33 (s, 18H). ^13^C NMR (101 MHz, Chloroform-*d*) δ 154.3, 147.2, 145.8, 136.6, 136.0, 135.0, 132.7, 132.1, 129.9, 129.8, 129.5, 128.9, 128.5, 128.1, 127.4, 126.3, 126.3, 125.4, 122.3, 120.4, 119.2, 111.9, 59.8, 34.4, 30.1, 21.9, 21.7. HRMS (ESI-TOF) calcd. for C_37_H_41_N_2_O_6_S [M + H]^+^ 641.2680; found: 641.2690.

**3k**, white solid, 41.8 mg, 69% yield; m.p. 178.6–179.4 °C; ^1^H NMR (400 MHz, Chloroform-*d*) δ 8.30 (d, *J* = 8.0 Hz, 1H), 7.83–7.76 (m, 2H), 7.71 (s, 1H), 7.69–7.60 (m, 1H), 7.50–7.27 (m, 6H), 7.24–7.16 (m, 2H), 7.07 (s, 1H), 6.82 (s, 2H), 6.70 (dd, *J* = 8.2, 1.6 Hz, 1H), 5.35 (s, 1H), 1.33 (s, 18H). ^13^C NMR (101 MHz, Chloroform-*d*) δ 154.4, 147.1, 136.7, 135.6, 135.5, 134.5, 132.0, 130.1, 129.9, 129.3, 128.8, 128.5, 128.1, 127.5, 126.1, 125.4, 124.8, 124.6, 122.5, 121.4, 120.8, 112.1, 60.0, 34.4, 30.2. HRMS (ESI-TOF) calcd. for C_35_H_36_N_2_O_6_SNa [M + Na]^+^ 635.2186; found: 635.2197.

**3l**, white solid, 33.9 mg, 66% yield; m.p. 168.4–169.1 °C; ^1^H NMR (400 MHz, Chloroform-*d*) δ 8.33 (d, *J* = 8.1 Hz, 1H), 7.82 (s, 1H), 7.45–7.33 (m, 2H), 7.36–7.25 (m, 2H), 7.20–7.11 (m, 2H), 6.90 (s, 2H), 6.85 (s, 1H), 6.60 (dd, *J* = 7.8, 1.6 Hz, 1H), 5.38 (s, 1H), 1.89 (s, 3H), 1.36 (s, 18H). ^13^C NMR (101 MHz, Chloroform-*d*) δ 168.2, 154.5, 148.1, 136.9, 135.5, 130.7, 130.3, 129.5, 128.9, 127.4, 126.4, 126.2, 125.6, 124.7, 124.5, 123.6, 121.3, 121.0, 111.6, 60.6, 34.4, 30.1, 20.3. HRMS (ESI-TOF) calcd. for C_31_H_34_N_2_O_5_Na [M + Na]^+^ 537.2360; found: 537.2369.

**3m**, white solid, 19.5 mg, 34% yield; m.p. 167.4–168.1 °C; ^1^H NMR (400 MHz, Chloroform-*d*) δ 8.31 (dt, *J* = 8.1, 1.0 Hz, 1H), 7.77 (s, 1H), 7.42–7.27 (m, 4H), 7.24 (dd, *J* = 8.1, 1.2 Hz, 1H), 7.16 (td, *J* = 7.6, 1.3 Hz, 1H), 7.02 (s, 1H), 6.89 (s, 2H), 6.63 (dd, *J* = 7.8, 1.6 Hz, 1H), 5.34 (s, 1H), 1.35 (s, 19H), 1.26 (s, 9H). ^13^C NMR (101 MHz, Chloroform-*d*) δ 154.5, 150.8, 148.4, 136.6, 135.5, 130.8, 130.4, 129.6, 128.8, 127.5, 126.5, 126.1, 125.7, 124.6, 124.5, 123.3, 121.4, 120.9, 111.7, 83.7, 59.9, 34.4, 30.1, 29.7, 27.4. HRMS (ESI-TOF) calcd. for C_34_H_40_N_2_O_6_Na [M + Na]^+^ 595.2779; found: 595.2790.

**3n**, white solid, 41.2 mg, 64% yield; m.p. 209.4–210.0 °C; ^1^H NMR (400 MHz, Chloroform-*d*) δ 8.24 (d, *J* = 8.0 Hz, 1H), 7.66–7.57 (m, 3H), 7.39–7.17 (m, 5H), 7.09 (dd, *J* = 9.1, 4.6 Hz, 1H), 6.95 (d, *J* = 9.3 Hz, 2H), 6.74 (s, 2H), 6.31 (dd, *J* = 8.7, 3.0 Hz, 1H), 5.30 (s, 1H), 2.36 (s, 3H), 1.27 (s, 18H). ^13^C NMR (101 MHz, Chloroform-*d*) δ 160.9 (d, *J* = 248.9 Hz), 154.6, 146.2, 142.8 (d, *J* = 3.1 Hz), 136.8, 135.4, 134.7 (d, *J* = 7.1 Hz), 132.3, 130.1, 129.7, 129.0, 128.2, 125.5, 124.9, 124.7, 124.3 (d, *J* = 8.7 Hz), 121.3, 120.9, 116.6 (d, *J* = 23.5 Hz), 115.4 (d, *J* = 25.1 Hz), 112.0, 60.1, 34.4, 30.1, 21.8. HRMS (ESI-TOF) calcd. for C_36_H_38_FN_2_O_6_S [M + H]^+^ 645.2429; found: 645.2437.

**3o**, white solid, 36.9 mg, 56% yield; m.p. 182.4–183.6 °C; ^1^H NMR (400 MHz, Chloroform-*d*) δ 8.32 (d, *J* = 8.0 Hz, 1H), 7.73–7.63 (m, 3H), 7.48–7.21 (m, 5H), 7.17 (d, *J* = 8.7 Hz, 1H), 7.01 (s, 1H), 6.81 (s, 2H), 6.67 (d, *J* = 2.6 Hz, 1H), 5.39 (s, 1H), 2.44 (s, 3H), 1.35 (s, 18H). ^13^C NMR (101 MHz, Chloroform-*d*) δ 154.6, 146.3, 145.6, 136.8, 135.4, 134.0, 133.2, 132.2, 130.1, 130.0, 129.7, 129.0, 128.3, 128.2, 125.4, 125.3, 124.9, 124.7, 123.8, 121.3, 120.9, 111.9, 59.9, 34.4, 30.1, 21.8. HRMS (ESI-TOF) calcd. for C_36_H_37_ClN_2_O_6_SNa [M + Na]^+^ 683.1953; found: 683.1961.

**3p**, white solid, 42.2 mg, 60% yield; m.p. 193.8–195.9 °C; ^1^H NMR (400 MHz, Chloroform-*d*) δ 8.32 (d, *J* = 8.0 Hz, 1H), 7.72–7.62 (m, 3H), 7.49–7.23 (m, 6H), 7.10 (d, *J* = 8.7 Hz, 1H), 7.00 (s, 1H), 6.82 (d, *J* = 2.3 Hz, 1H), 6.80 (s, 2H), 5.38 (s, 1H), 2.43 (s, 3H), 1.34 (s, 18H). ^13^C NMR (101 MHz, Chloroform-*d*) δ 154.6, 146.3, 146.2, 136.8, 135.4, 134.3, 133.0, 132.2, 131.2, 130.1, 129.7, 129.1, 128.2, 125.4, 125.4, 124.9, 124.7, 124.1, 121.3, 120.9, 111.9, 59.8, 3.4, 30.1, 21.8. HRMS (ESI-TOF) calcd. for C_36_H_37_BrN_2_O_6_SNa [M + Na]^+^ 729.1434; found: 729.1448.

**3q**, white solid, 38.0 mg, 54% yield; m.p. 259.8–260.4 °C; ^1^H NMR (400 MHz, Chloroform-*d*) δ 8.30 (d, *J* = 8.0 Hz, 1H), 7.70–7.63 (m, 3H), 7.45–7.37 (m, 2H), 7.37–7.21 (m, 6H), 6.96 (s, 1H), 6.79 (s, 2H), 6.55 (d, *J* = 8.3 Hz, 1H), 5.37 (s, 1H), 2.44 (s, 3H), 1.33 (s, 18H). ^13^C NMR (101 MHz, Chloroform-*d*) δ 154.5, 147.2, 146.3, 136.8, 135.4, 132.1, 131.2, 130.6, 130.1, 129.8, 129.5, 128.9, 128.2, 125.8, 125.5, 125.3, 124.9, 124.7, 122.7, 121.3, 120.8, 112.0, 59.8, 34.4, 30.1, 21.8. HRMS (ESI-TOF) calcd. for C_36_H_37_BrN_2_O_6_SNa [M + Na]^+^ 729.1434; found: 729.1439.

**3r**, white solid, 45.4 mg, 71% yield; m.p. 196.3–197.4 °C; ^1^H NMR (400 MHz, Chloroform-*d*) δ 8.34–8.27 (m, 1H), 7.72 (s, 1H), 7.69–7.62 (m, 2H), 7.45–7.36 (m, 2H), 7.36–7.20 (m, 4H), 7.11 (dd, *J* = 8.4, 1.9 Hz, 1H), 7.06 (d, *J* = 17.7 Hz, 2H), 6.80 (s, 2H), 6.49 (d, *J* = 1.9 Hz, 1H), 5.33 (s, 1H), 2.42 (s, 3H), 2.21 (s, 3H), 1.33 (s, 18H). ^13^C NMR (101 MHz, Chloroform-*d*) δ 154.3, 145.8, 145.0, 137.5, 136.6, 135.6, 132.7, 131.5, 130.4, 130.2, 129.9, 128.8, 128.7, 128.2, 126.3, 125.4, 124.7, 124.5, 122.2, 121.3, 120.7, 112.2, 59.9, 34.4, 30.2, 21.8, 21.2. HRMS (ESI-TOF) calcd. for C_37_H_40_N_2_O_6_SNa [M + Na]^+^ 663.2499; found: 663.2508.

**3s,** white solid, 28.9 mg, 44% yield; m.p. 203.5–204.6 °C; ^1^H NMR (400 MHz, Chloroform-*d*) δ 8.29 (d, *J* = 8.0 Hz, 1H), 7.67 (s, 1H), 7.61 (d, *J* = 8.0 Hz, 2H), 7.44–7.36 (m, 1H), 7.29 (dd, *J* = 16.3, 9.0 Hz, 2H), 7.19 (d, *J* = 8.0 Hz, 2H), 6.96 (s, 1H), 6.86 (d, *J* = 2.5 Hz, 1H), 6.76 (s, 2H), 6.73 (d, *J* = 2.5 Hz, 1H), 6.65 (d, *J* = 8.7 Hz, 1H), 5.31 (s, 1H), 3.76 (s, 3H), 2.40 (s, 3H), 1.32 (s, 18H). ^13^C NMR (101 MHz, Chloroform-*d*) δ 160.5, 154.1, 148.0, 145.9, 136.5, 135.5, 132.4, 130.0, 129.9, 129.6, 128.6, 128.1, 126.7, 124.9, 124.7, 124.5, 123.3, 121.3, 120.7, 112.9, 112.2, 108.3, 59.4, 55.7, 34.4, 30.2, 21.8. HRMS (ESI-TOF) calcd. for C_37_H_40_N_2_O_7_SNa [M + Na]^+^ 679.2448; found: 679.2458.

**3t**, white solid, 48.5 mg, 74% yield; m.p. 216.9–217.6 °C; ^1^H NMR (400 MHz, Chloroform-*d*) δ 8.30 (d, *J* = 8.1 Hz, 1H), 7.87–7.80 (m, 3H), 7.54 (d, *J* = 8.3 Hz, 1H), 7.43–7.35 (m, 1H), 7.35–7.27 (m, 3H), 7.21 (s, 1H), 7.18–7.08 (m, 1H), 6.90 (s, 3H), 6.31 (dd, *J* = 8.0, 1.4 Hz, 1H), 5.34 (s, 1H), 3.59 (s, 3H), 2.44 (s, 3H), 1.35 (s, 18H). ^13^C NMR (101 MHz, Chloroform-*d*) δ 154.4, 152.6, 145.2, 136.7, 136.5, 135.7, 134.6, 134.2, 130.3, 129.5, 128.7, 128.2, 127.9, 126.1, 125.8, 124.7, 124.5, 121.4, 120.7, 119.2, 112.7, 112.5, 60.5, 55.6, 34.4, 30.2, 21.7. HRMS (ESI-TOF) calcd. for C_37_H_40_N_2_O_7_SNa [M + Na]^+^ 679.2448; found: 679.2455.

### 3.3. The Experimental Procedure for Synthesis of Compound **6** (Scheme 5)

In a reaction tube equipped with a magnetic stirring bar, the indole-3-carboxylate **5** (0.1 mmol, 1 equiv), *ortho*-tosylaminophenyl *p*-QMs **2** (0.1 mmol, 1.0 equiv), K_2_CO_3_ (0.2 mmol, 2.0 equiv) and acetonitrile (1.0 mL) were added. Then, the mixture was stirred at room temperature. After completion, the mixture was concentrated and purified by flash chromatography on silica gel (petroleum ether/ethyl acetate = 15/1) to afford **6** in 40% yield.

**6**, 25.5 mg, 40% yield; ^1^H NMR (400 MHz, Chloroform-*d*) δ 8.19–8.14 (m, 1H), 7.68–7.62 (m, 2H), 7.48 (s, 1H), 7.31–7.26 (m, 2H), 7.26–7.24 (m, 2H), 7.23–7.21 (m, 1H), 7.21–7.17 (m, 2H), 7.17–7.13 (m, 1H), 6.99 (s, 1H), 6.78 (s, 2H), 6.65 (dd, J = 7.8, 1.6 Hz, 1H), 5.27 (s, 1H), 3.88 (s, 3H), 2.41 (s, 3H), 1.32 (s, 18H). ^13^C NMR (101 MHz, Chloroform-*d*) δ 165.7, 153.9, 147.2, 145.6, 136.9, 136.2, 133.6, 132.9, 132.8, 129.9, 129.4, 128.7, 128.1, 127.3, 127.2, 126.9, 125.5, 122.9, 122.2, 122.1, 121.5, 111.4, 107.1, 59.3, 51.0, 34.3, 30.2, 21.7. HRMS (ESI-TOF) calcd. for C_38_H_41_NO_6_SNa [M + Na]^+^ 662.2547; found: 662.2553.

### 3.4. The Experimental Procedure for Synthesis of Compound **11** (Scheme 5)

In a reaction tube equipped with a magnetic stirring bar, the 3-nitroindoles **1** (0.1 mmol, 1 equiv), *ortho*-OMe phenyl p-QM **10** (0.1 mmol, 1.0 equiv), K_2_CO_3_ (0.2 mmol, 2.0 equiv), PhOH (1.0 mmol, 1.0 equiv) and acetonitrile (1.0 mL) were added. Then, the mixture was stirred at room temperature. After completion, the mixture was concentrated and purified by flash chromatography on silica gel to give the corresponding product **11**.

**11**, white solid, 9.7 mg, 20% yield; m.p. 227.4–228.2 °C; ^1^H NMR (400 MHz, Chloroform-*d*) δ 8.30 (d, *J* = 8.1 Hz, 1H), 7.83 (s, 1H), 7.43–7.22 (m, 4H), 7.10 (s, 1H), 6.99–6.89 (m, 4H), 6.78 (dd, *J* = 7.7, 1.7 Hz, 1H), 5.30 (s, 1H), 3.77 (s, 3H), 1.35 (s, 18H). ^13^C NMR (101 MHz, Chloroform-*d*) δ 156.7, 154.0, 136.4, 135.7, 130.4, 130.0, 128.6, 128.4, 127.1, 126.4, 125.1, 124.3, 124.3, 121.4, 120.9, 120.8, 111.9, 111.0, 59.2, 55.7, 34.4, 30.2. HRMS (ESI-TOF) calcd. for C_30_H_34_N_2_O_4_Na [M + Na]^+^ 509.2411; found: 509.2419.

## 4. Conclusions

In conclusion, we have described an unprecedented *N*-alkylation of 3-nitroindoles and *para*-quinone methides by using K_2_CO_3_ was the base via a protection group migration/*aza*-1,6-Michael addition sequences. With the developed protocol, a series of structurally diverse *N*-diarylmethylindole derivatives were obtained in moderate to good yields under mild conditions. According to the control experiments, a reasonable reaction mechanism was proposed. Importantly, the reaction herein is featured that 3-nitrodoles is defined with latent *N*-centered nucleophiles to react with *ortho*-hydrophenyl *p*-QMs, which is different from the previous reports where 3-nitrodoles was served as electrophiles trapped by various nucleophiles.

## Figures and Tables

**Table 1:** Optimization of reaction conditions [a].

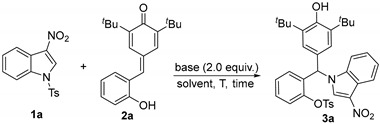					
Entry	Base	Solvent	T (°C)	Time (h)	Yield [b]
**1**	DABCO	toluene	50	168	44
**2**	DBU	toluene	50	68	24
**3**	Na_2_CO_3_	toluene	50	145	trace
**4**	K_2_CO_3_	toluene	50	26	60
**5**	K_2_CO_3_	CH_2_Cl_2_	50	88	57
**6**	K_2_CO_3_	THF	50	63	58
**7**	K_2_CO_3_	EtOAc	50	136	63
**8**	K_2_CO_3_	CH_3_CN	50	23	67
**9**	K_2_CO_3_	MeOH	50	20	19
**10**	K_2_CO_3_	CH_3_CN	rt	23	71
**11** [c]	K_2_CO_3_	CH_3_CN	rt	48	64

[a] Unless otherwise noted, the reaction was carried out with **1a** (0.05 mmol), **2a** (0.05 mmol), and base (2.0 equiv.) in 0.5 mL of solvent at indicated temperature for specified time. [b] Isolated yield. [c] 1.0 equiv K_2_CO_3_ was used.

## Data Availability

All the required data are reported in the manuscript and Supplementary Materials.

## Supplementary Materials

The following supporting information can be downloaded at: https://www.mdpi.com/article/10.3390/molecules28145529/s1, X-ray data for products **3l** and **3p**; copies of ^1^H and ^13^C NMR spectra.

## Sample Availability

Samples of the compounds are available from the authors upon request.
