# Supplementary material for: 3-Nitroindoles Serving as N-Centered Nucleophiles for Aza-1,6-Michael Addition to para-Quinone Methides
Source: Molecules. 2023 Jul 20;28(14):5529. doi: 10.3390/molecules28145529 (PMC10384903; doi:10.3390/molecules28145529)

3-Nitroindoles Serving as *N*-Centered Nucleophiles for Aza-1,6-Michael Addition to *para*-Quinone Methides

Jian-Qiang Zhao[1,]*, Wen-Jie Wang[1], Shun Zhou[1,2], Qi-Lin Xiao[1], Xi-Sha Xue[1], Yan-Ping Zhang[1], Yong You [1], Zhen-Hua Wang[1], and Wei-Cheng Yuan[1,]*

1. Innovation Research Center of Chiral Drugs, Institute for Advanced Study, Chengdu University, Chengdu 610106, China
2. National Engineering Research Center of Chiral Drugs, Chengdu Institute of Organic Chemistry, Chinese Academy of Sciences, Chengdu 610041

Correspondence: zhaojianqiang@cdu.edu.cn (J.-Q. Zhao);
yuanwc@cioc.ac.cn (W.-C. Yuan)

Supporting Information

Table of Contents

1. X-Ray crystal data for compound **3l** and **3p**..S1
2. ^{1}H, and ^{13}C NMR spectra for compounds **3, 6 and 11**...S3

1. X-Ray crystal data for compounds **3l and 3p**.

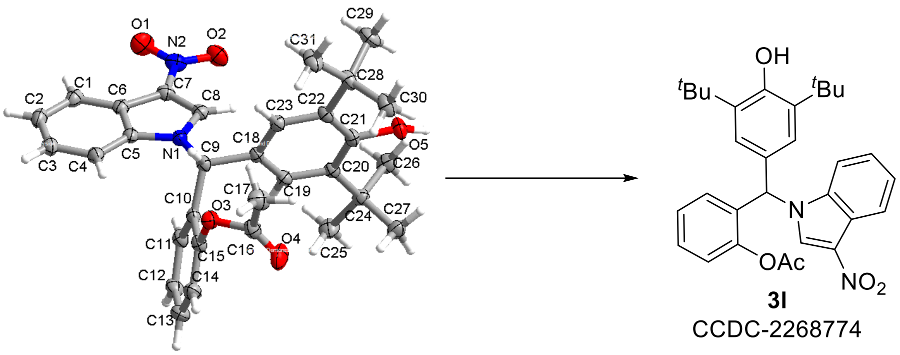

3l
CCDC-2268774

ORTEP of **3l** (at 50% level)

Table 1 Crystal data and structure refinement for 3l

Identification code	**3l**
Empirical formula	$C_{31}H_{34}N_2O_5$
Formula weight	514.60
Temperature/K	293(2)
Crystal system	monoclinic
Space group	$P2_1/c$
a/Å	11.4957(2)
b/Å	22.7104(4)
c/Å	11.06142(18)
α/°	90
β/°	105.5630(18)
γ/°	90
Volume/Å^3	2781.95(9)
Z	4
ρ_{calc}g/cm^3	1.229
μ/mm^{-1}	0.672
F(000)	1096.0
Crystal size/mm^3	0.15 × 0.12 × 0.1
Radiation	CuKα (λ = 1.54184)
2Θ range for data collection/°	7.786 to 140.926
Index ranges	-9 ≤ h ≤ 13, -27 ≤ k ≤ 26, -13 ≤ l ≤ 11
Reflections collected	10598
Independent reflections	5213 [R_{int} = 0.0319, R_{sigma} = 0.0467]
Data/restraints/parameters	5213/0/351
Goodness-of-fit on F^2	1.027
Final R indexes [I>=2σ (I)]	R_1 = 0.0520, wR_2 = 0.1360
Final R indexes [all data]	R_1 = 0.0666, wR_2 = 0.1519
Largest diff. peak/hole / e Å^{-3}	0.30/-0.30

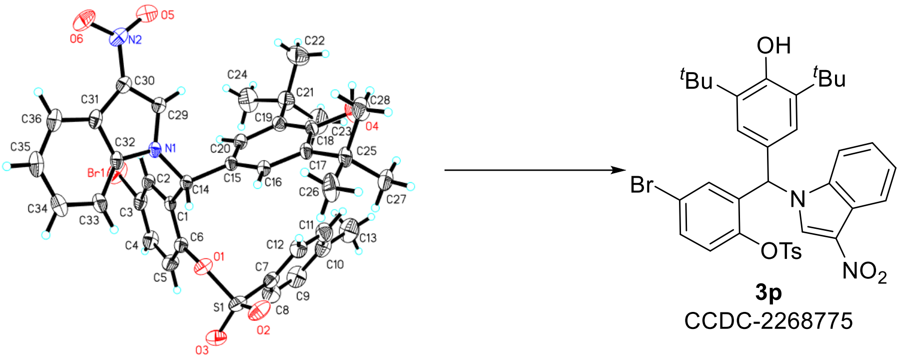

3p
CCDC-2268775

ORTEP of **3p** (at 50% level)

Table 1 Crystal data and structure refinement for 3p

Identification code	3p
Empirical formula	$C_{36}H_{37}BrN_2O_6S$
Formula weight	705.64
Temperature/K	249.99(10)
Crystal system	monoclinic
Space group	$P2_1/c$
a/Å	11.18050(10)
b/Å	16.1823(2)
c/Å	21.0128(2)
α/°	90
β/°	95.2830(10)
γ/°	90
Volume/$Å^3$	3785.62(7)
Z	4
$\rho_{calc}g/cm^3$	1.238
μ/mm^{-1}	2.346
F(000)	1464.0
Crystal size/mm^3	0.13 × 0.12 × 0.1
Radiation	Cu Kα (λ = 1.54184)
2Θ range for data collection/°	6.906 to 143.3
Index ranges	$-13 \le h \le 9, -19 \le k \le 15, -25 \le l \le 25$
Reflections collected	22216
Independent reflections	7234 [R_{int} = 0.0438, R_{sigma} = 0.0404]
Data/restraints/parameters	7234/0/423
Goodness-of-fit on F^2	1.070
Final R indexes [I>=2σ (I)]	R_1 = 0.0482, wR_2 = 0.1367
Final R indexes [all data]	R_1 = 0.0529, wR_2 = 0.1403
Largest diff. peak/hole / e $Å^{-3}$	0.68/-0.75

2. ^{1}H and ^{13}C NMR spectra for compounds **3, 6,** and **11**

^{1}H NMR spectrum of 3a

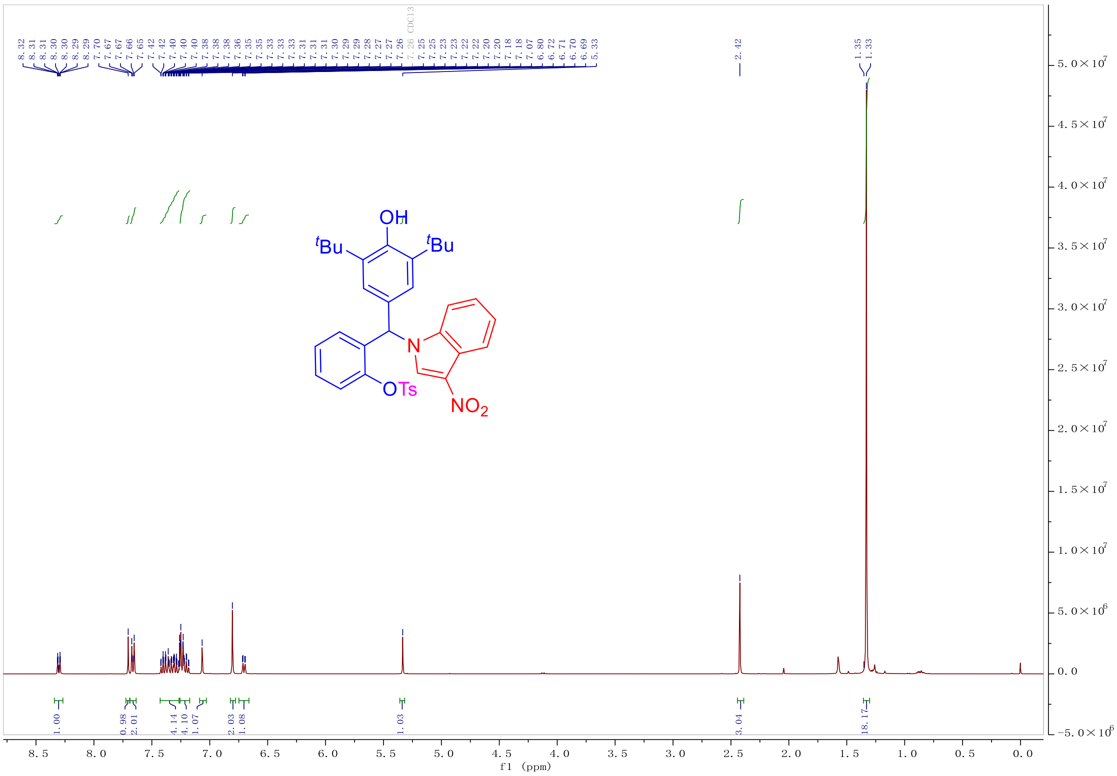

^{13}C NMR spectrum of 3a

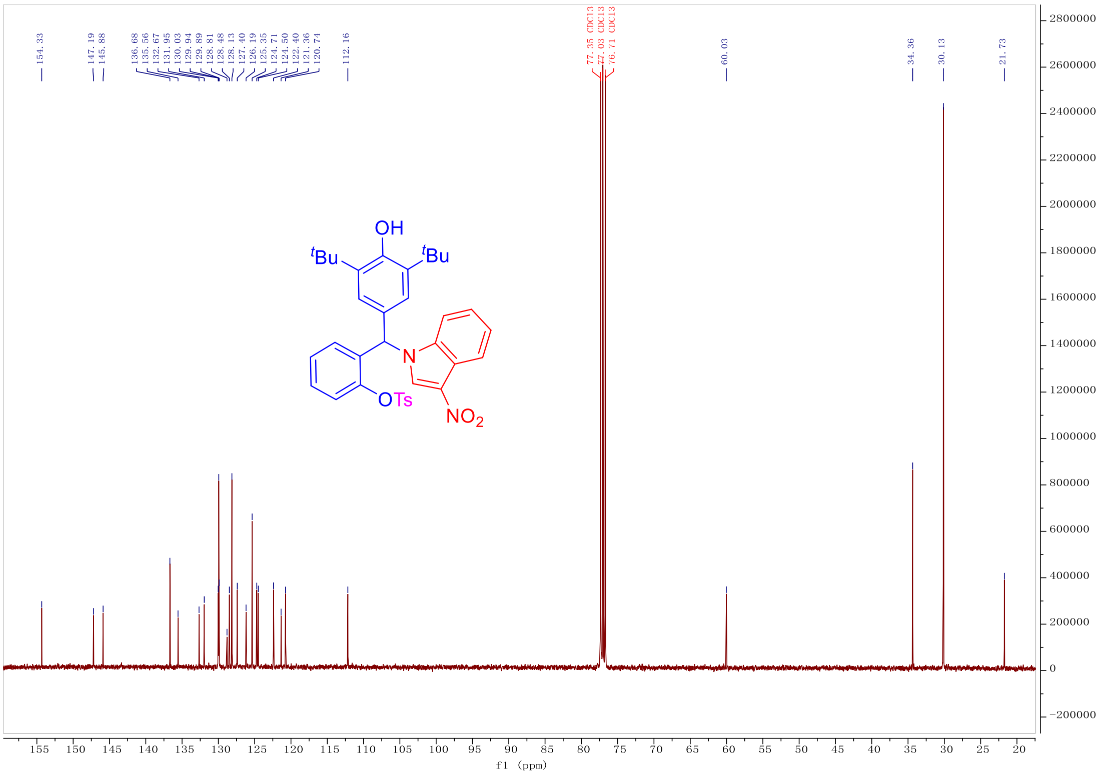

^{1}H NMR spectrum of 3b

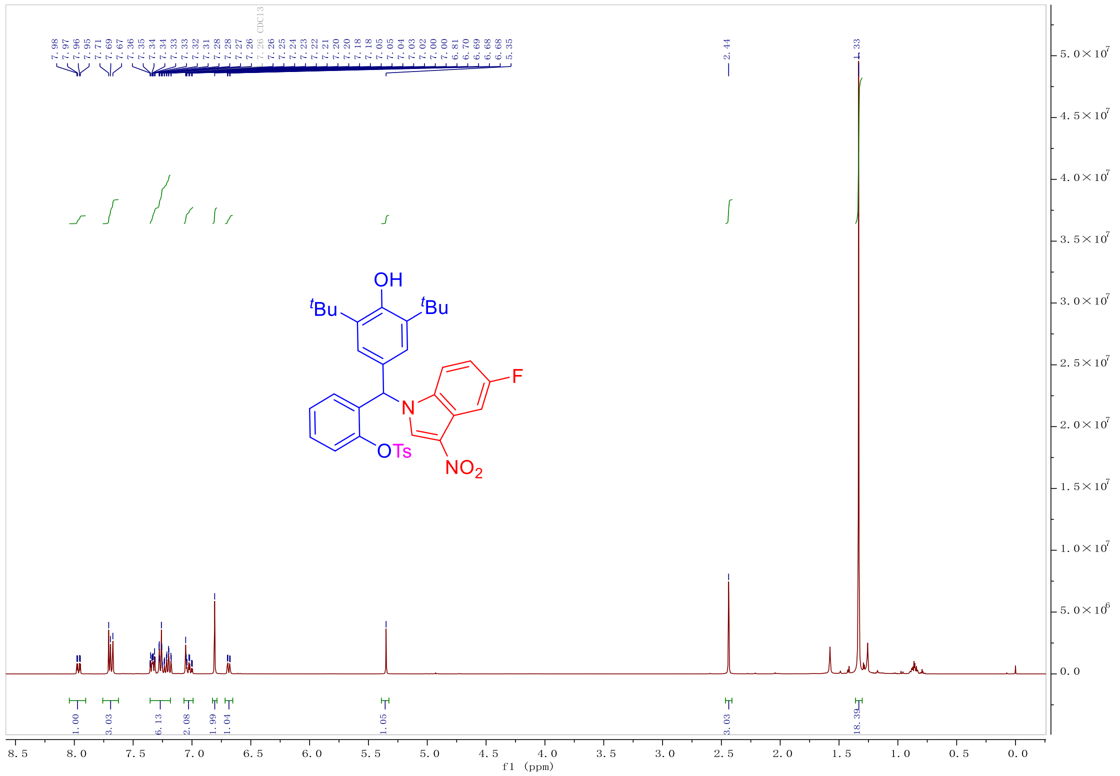

^{13}C NMR spectrum of 3b

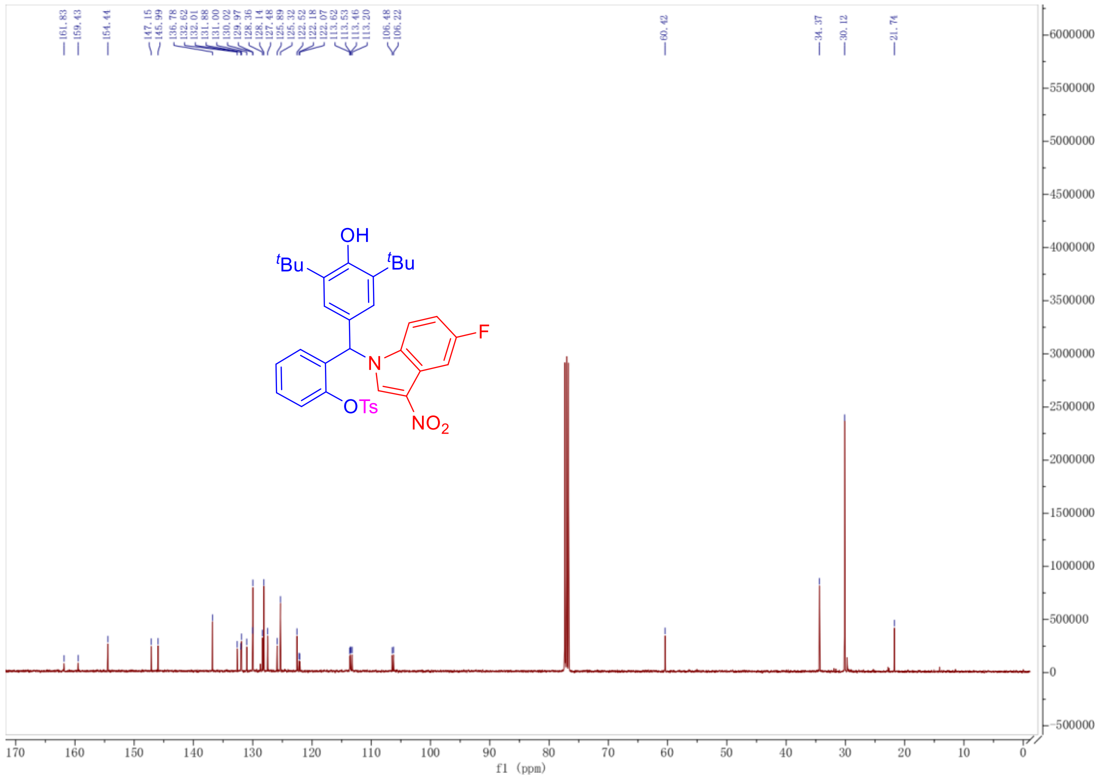

^{1}H NMR spectrum of 3c

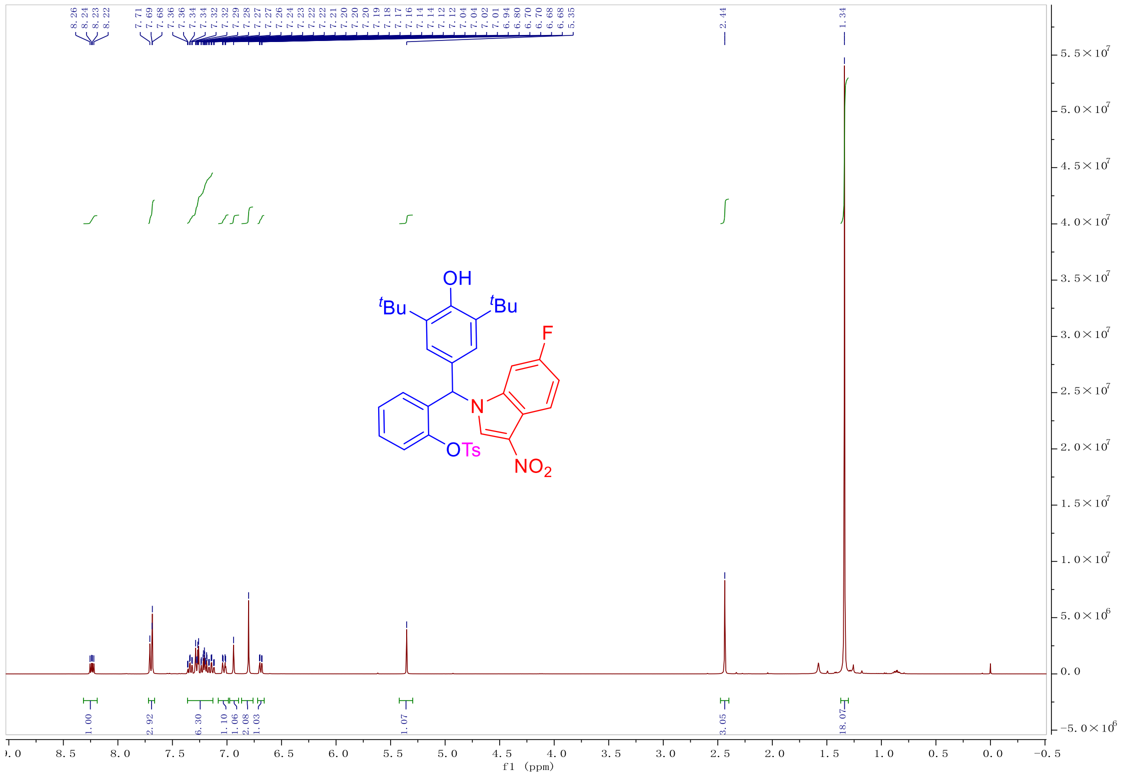

^{13}C NMR spectrum of 3c

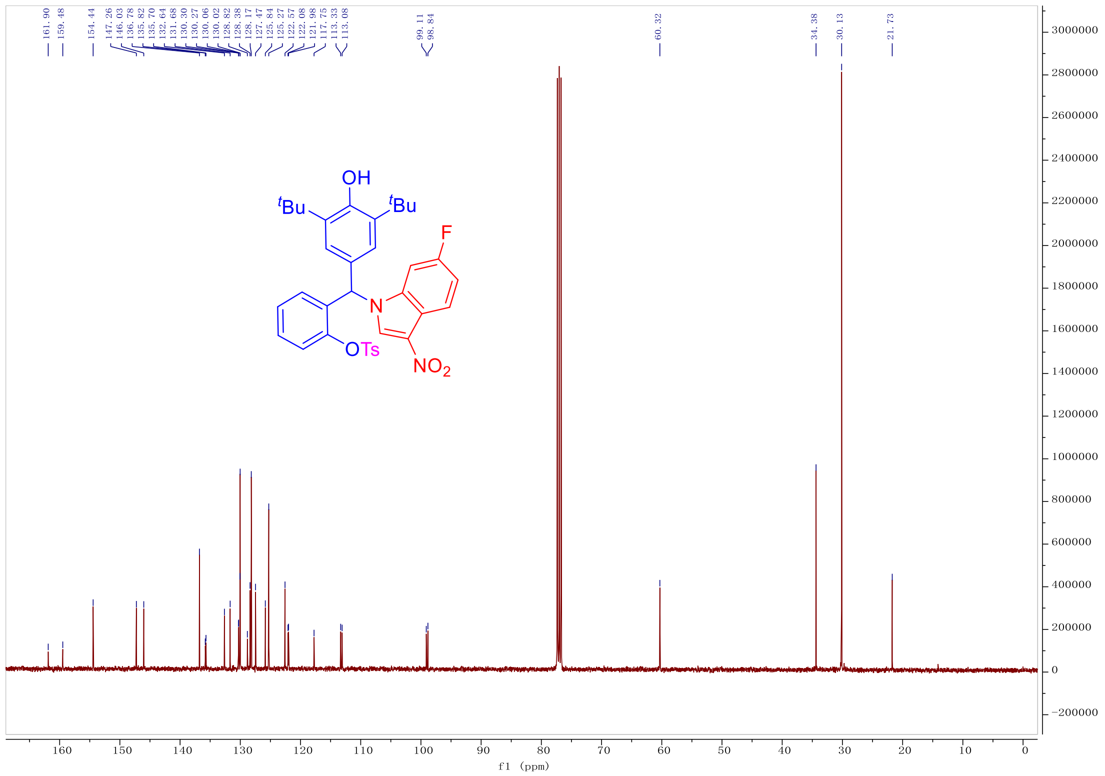

^{1}H NMR spectrum of 3d

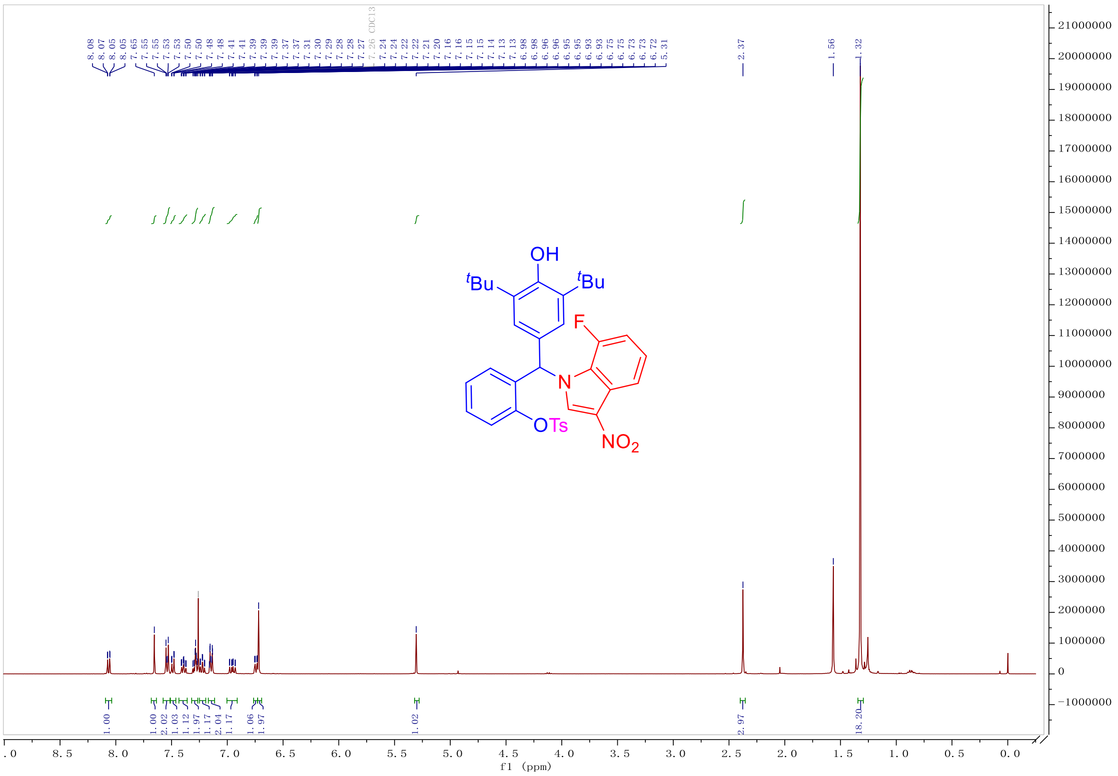

^{13}C NMR spectrum of 3d

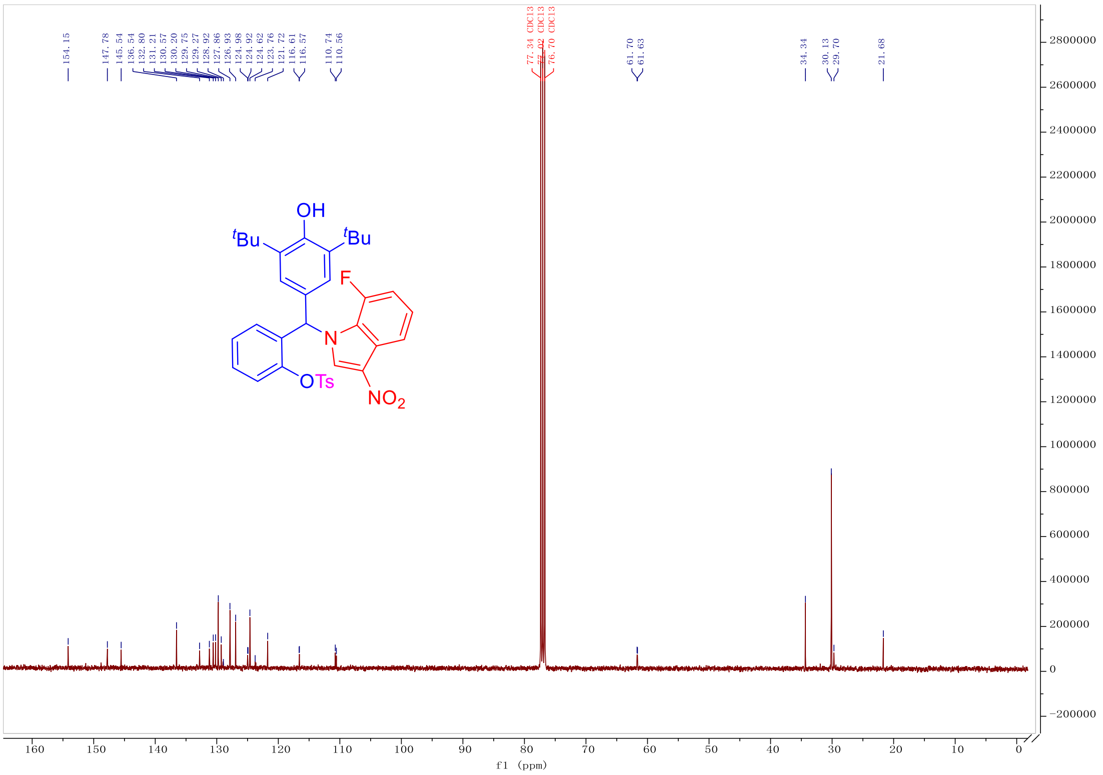

^{1}H NMR spectrum of 3e

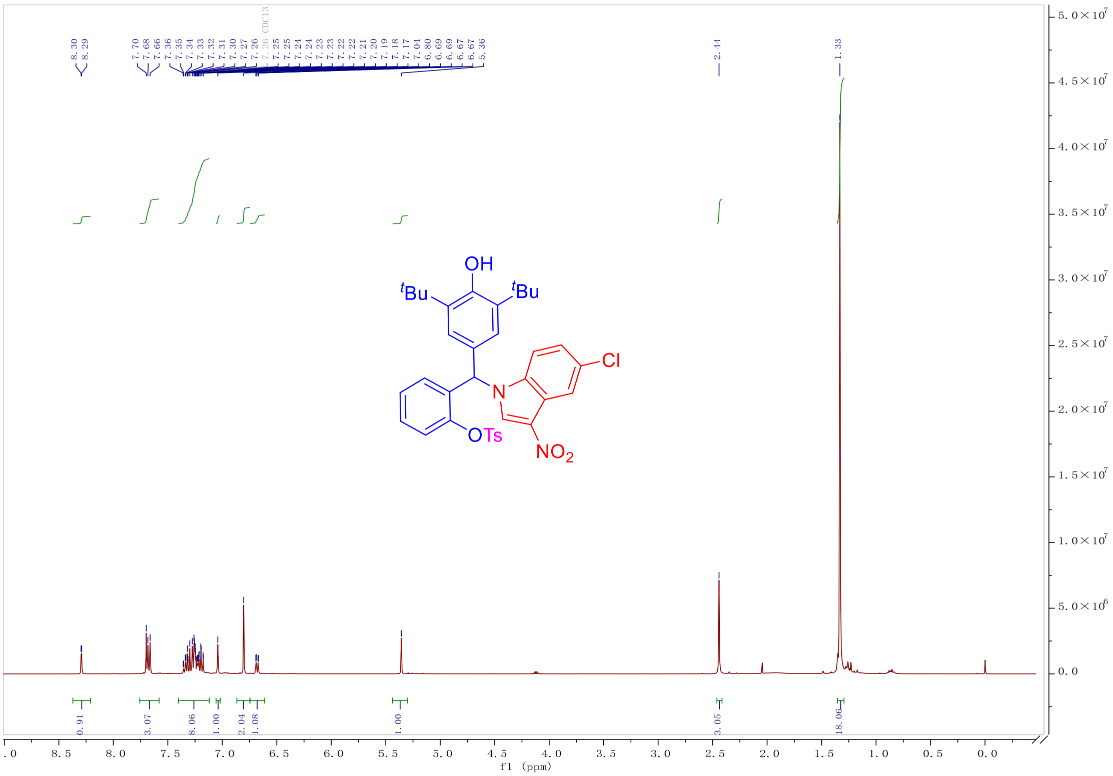

^{13}C NMR spectrum of 3e

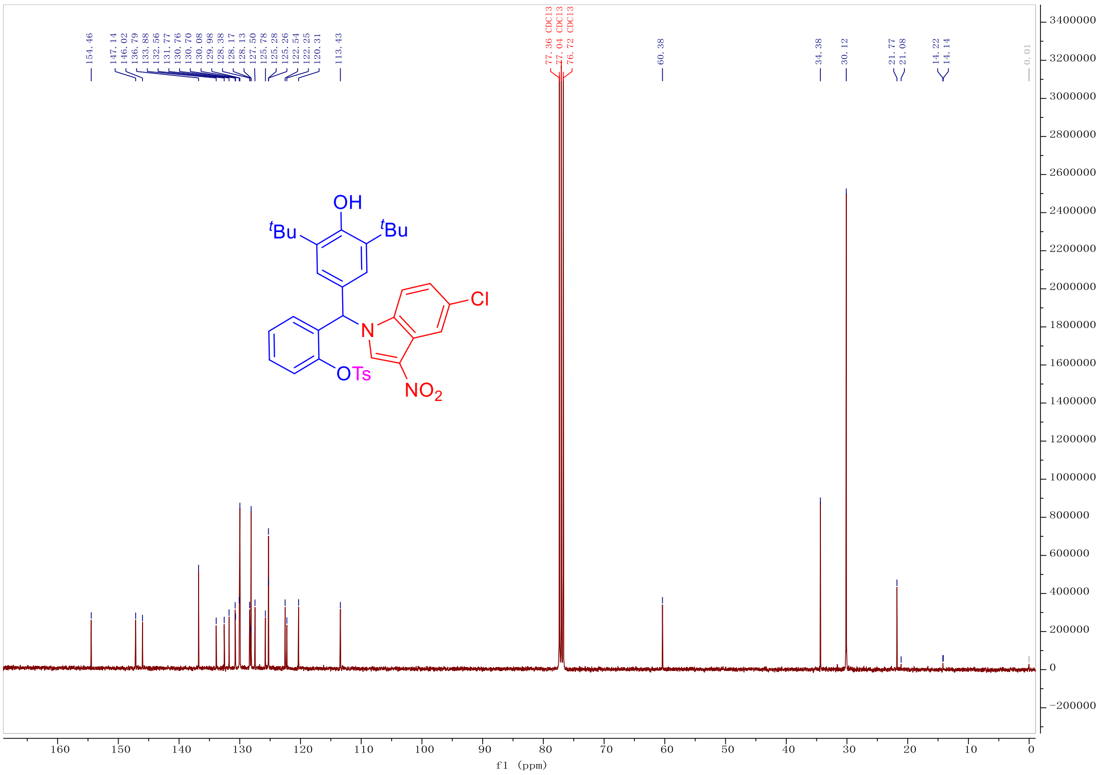

^{1}H NMR spectrum of 3f

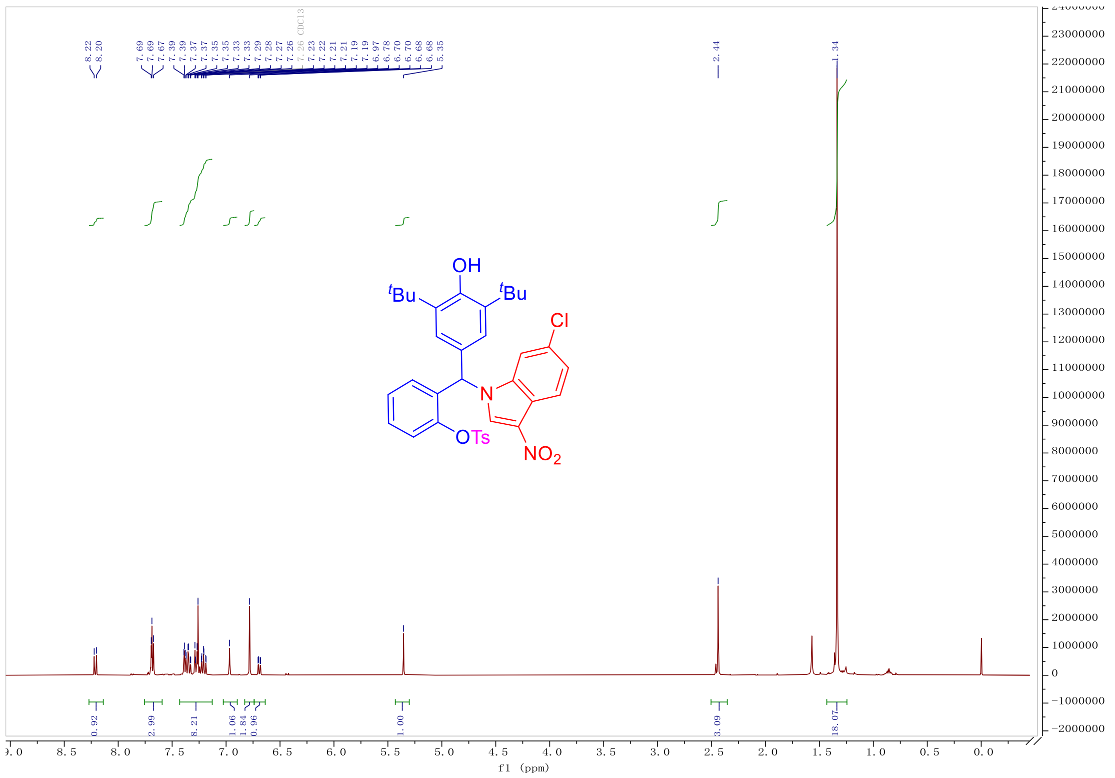

^{13}C NMR spectrum of 3f

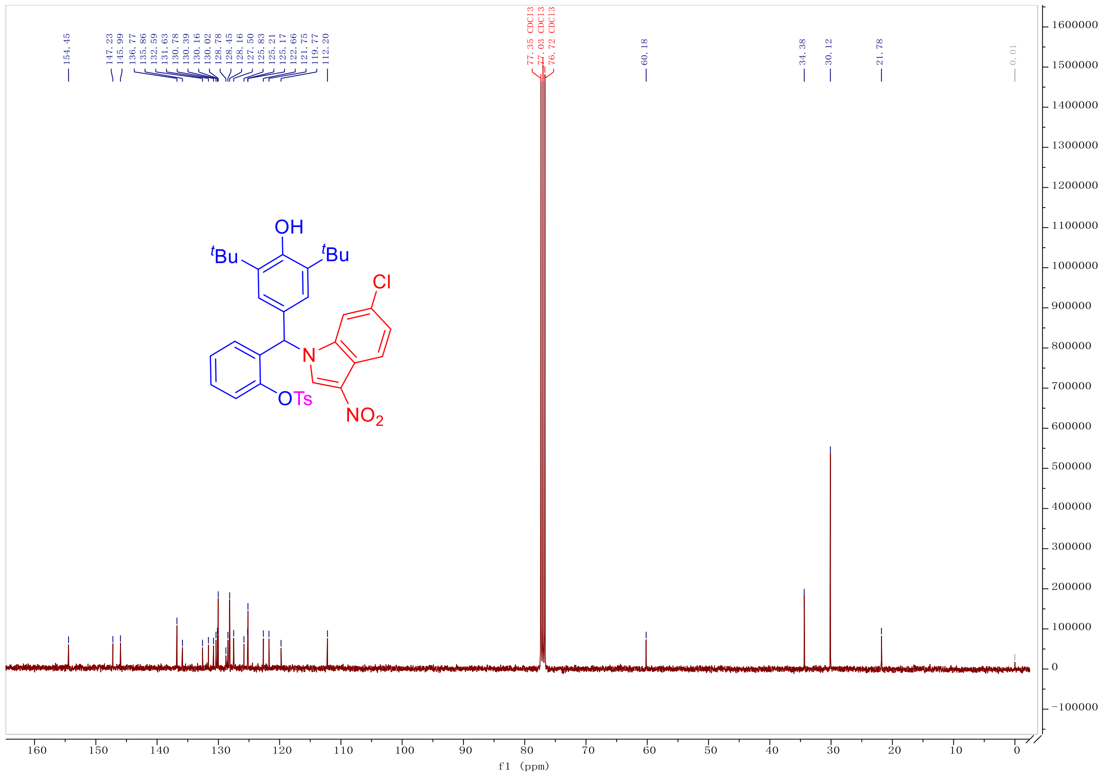

^{1}H NMR spectrum of 3g

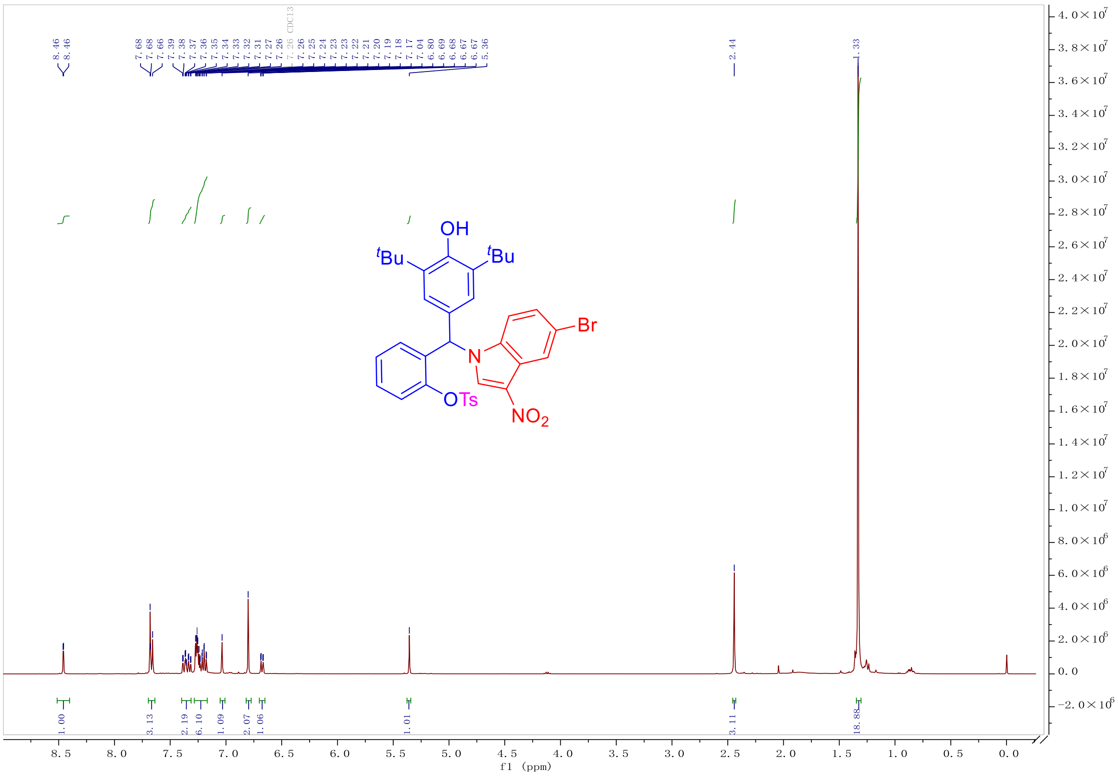

^{13}C NMR spectrum of 3g

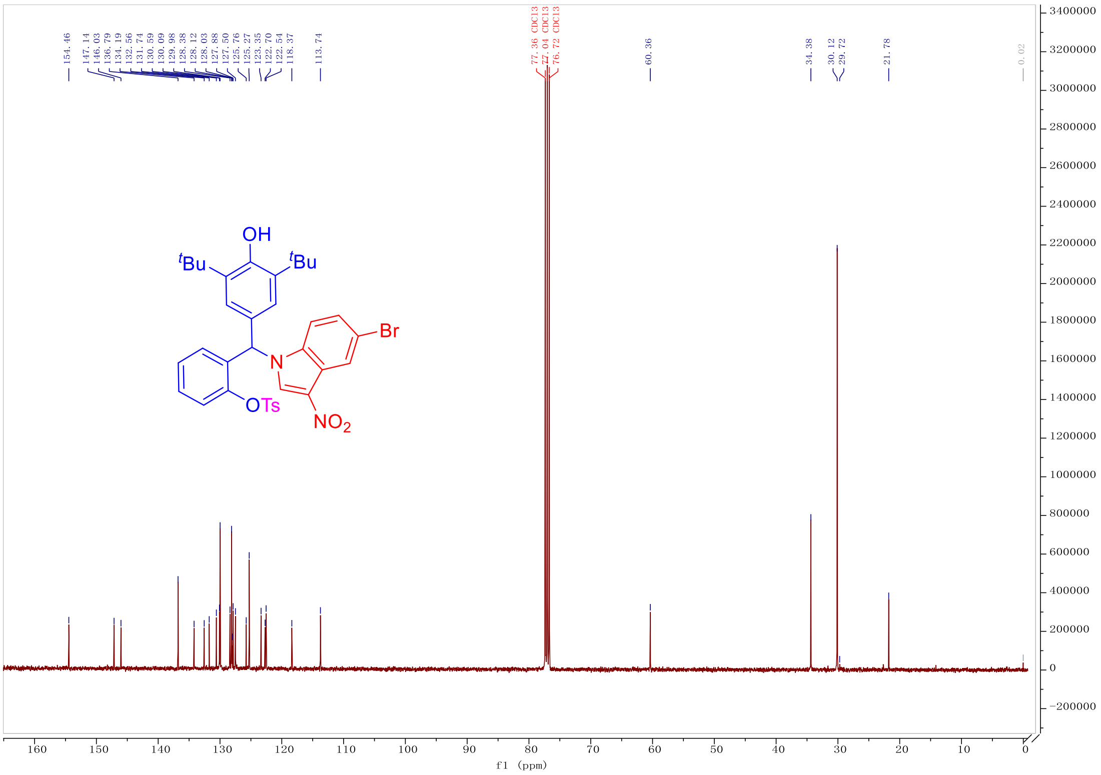

^{1}H NMR spectrum of 3h

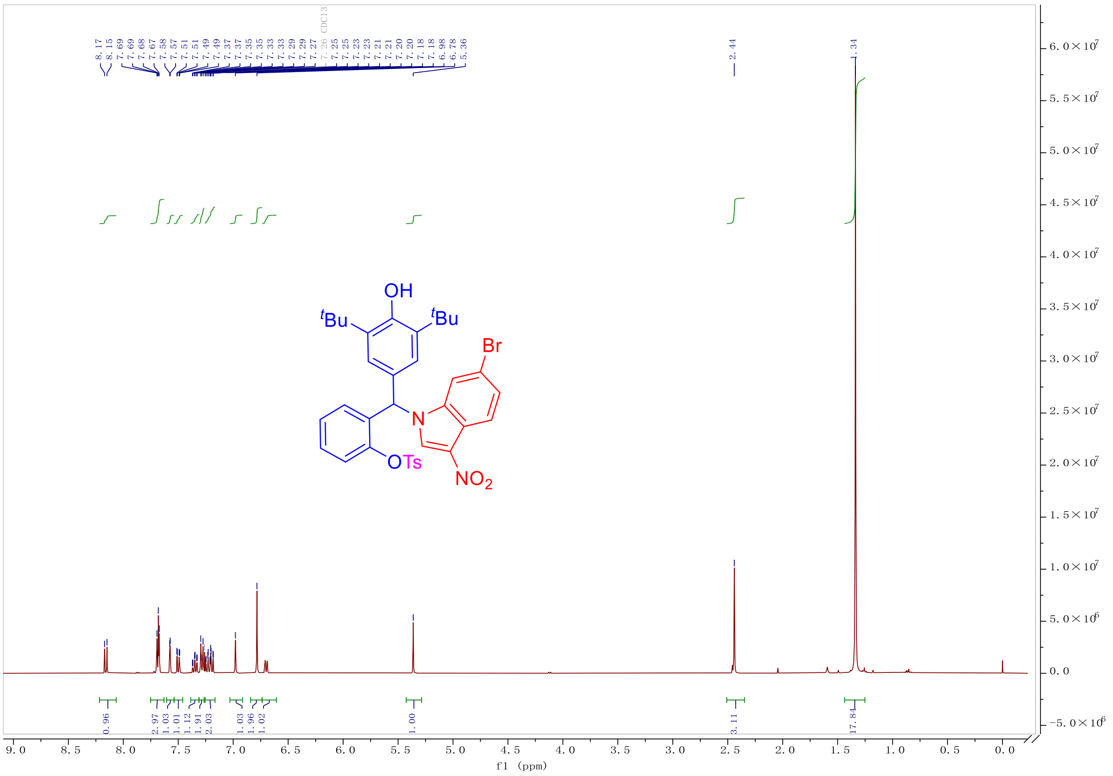

^{13}C NMR spectrum of 3h

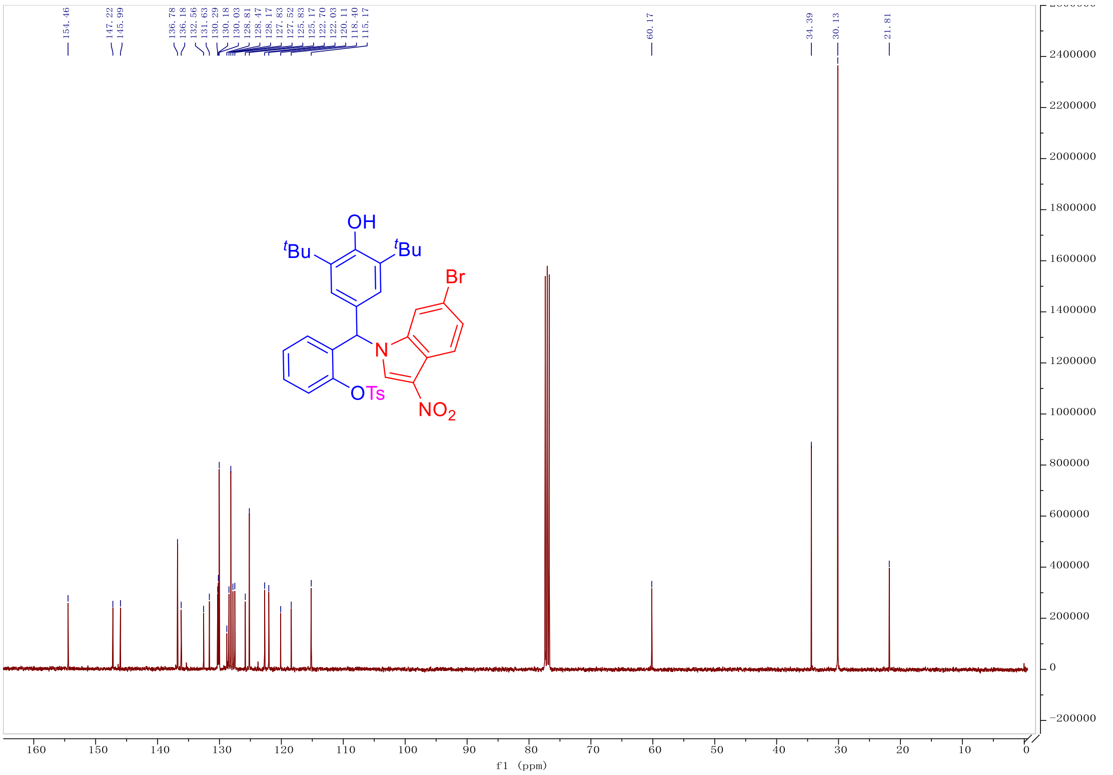

^{1}H NMR spectrum of 3i

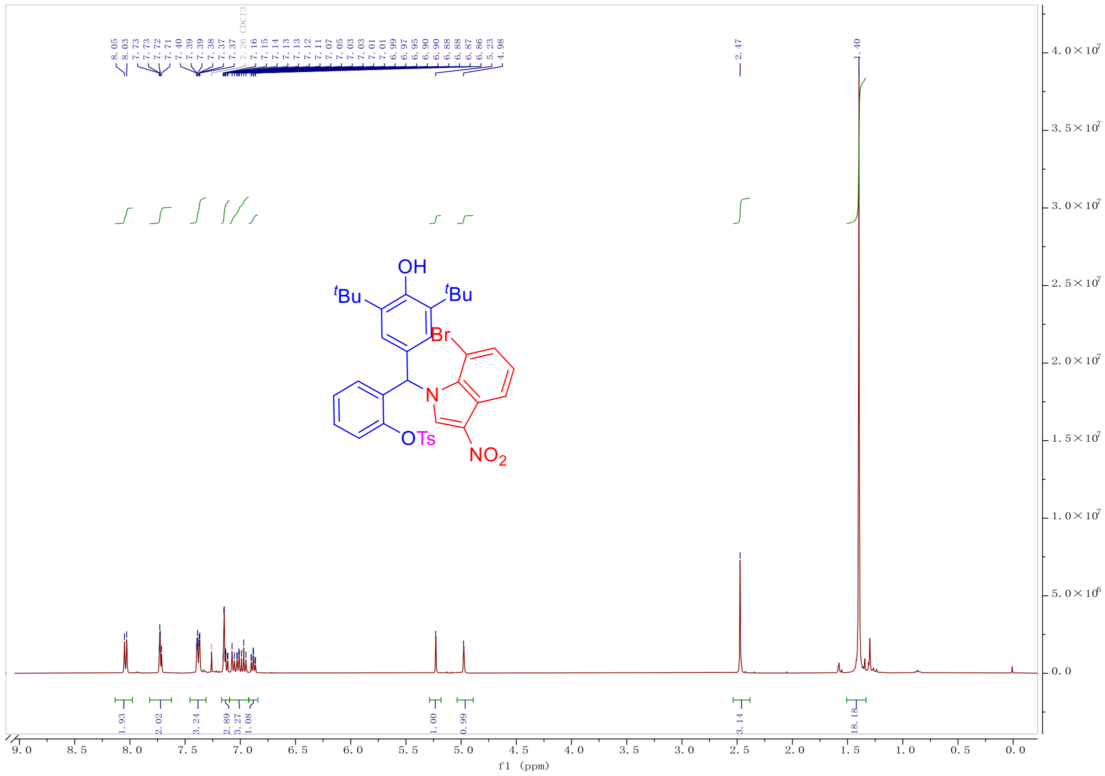

^{13}C NMR spectrum of 3i

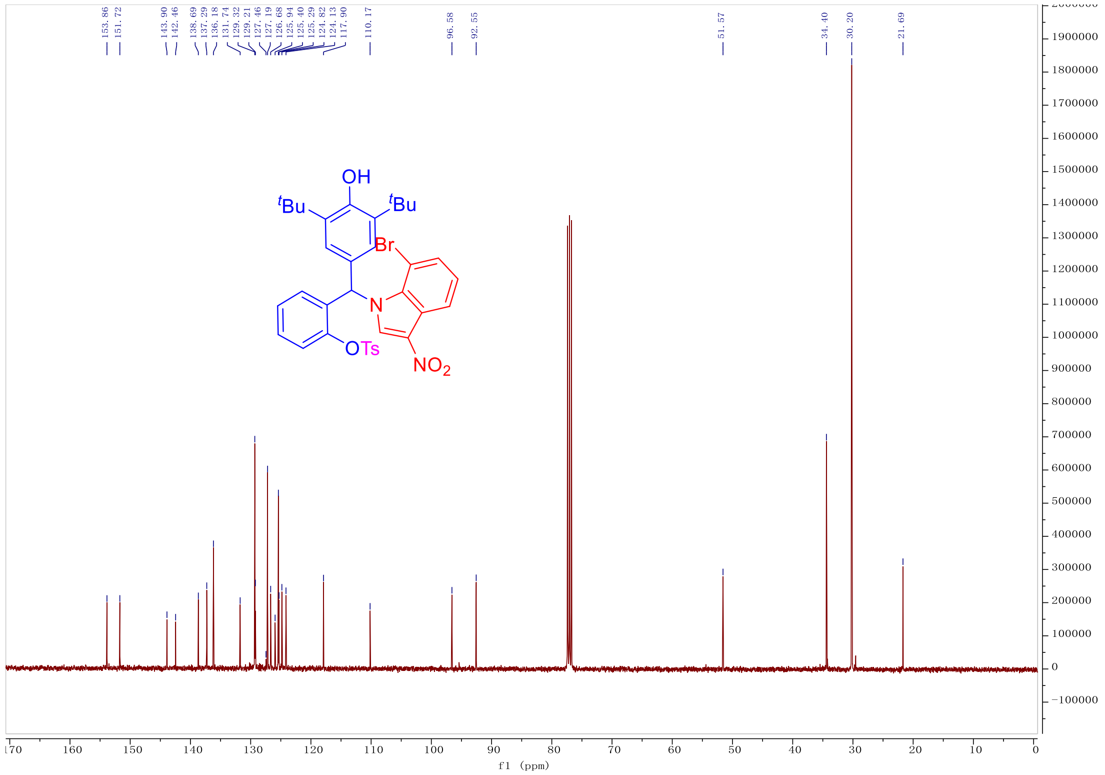

^{1}H NMR spectrum of 3j

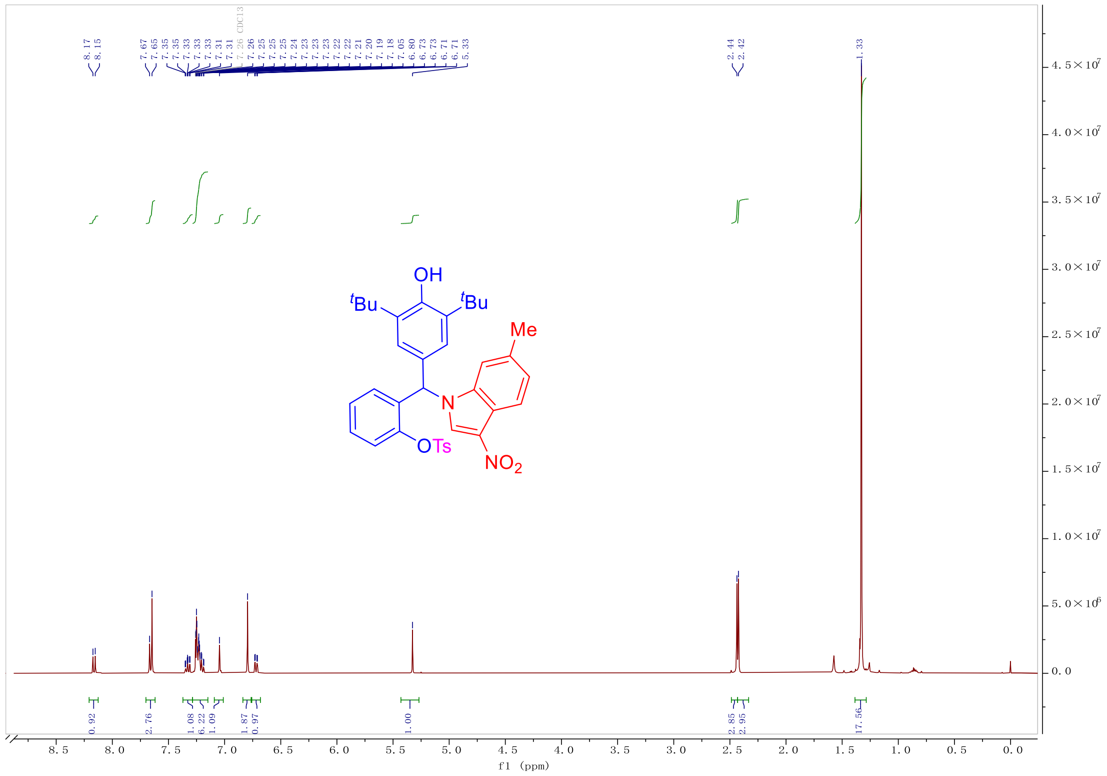

^{13}C NMR spectrum of 3j

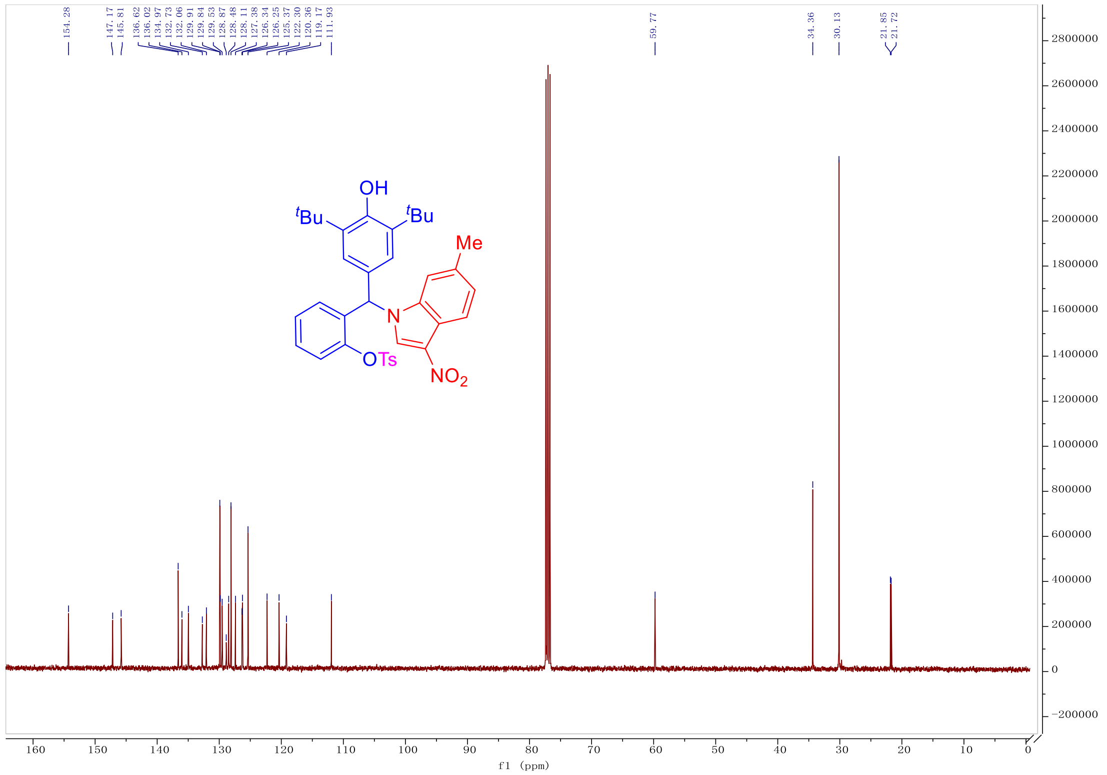

^{1}H NMR spectrum of 3k

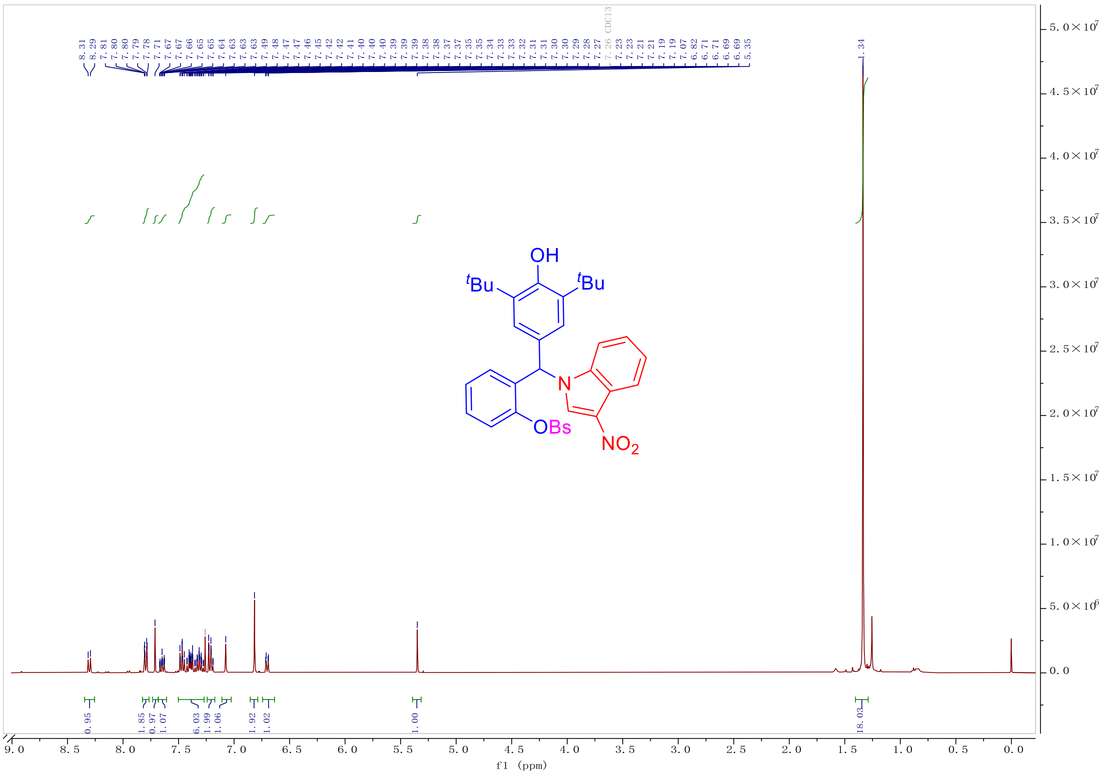

^{13}C NMR spectrum of 3k

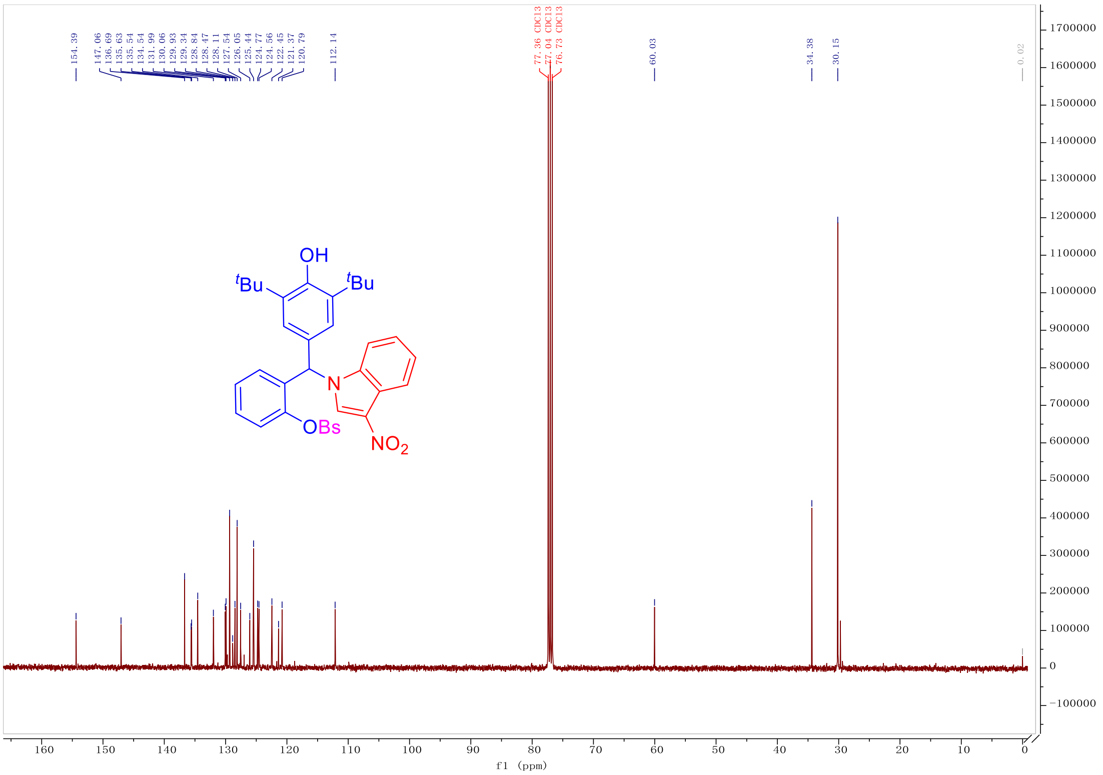

^{1}H NMR spectrum of 3l

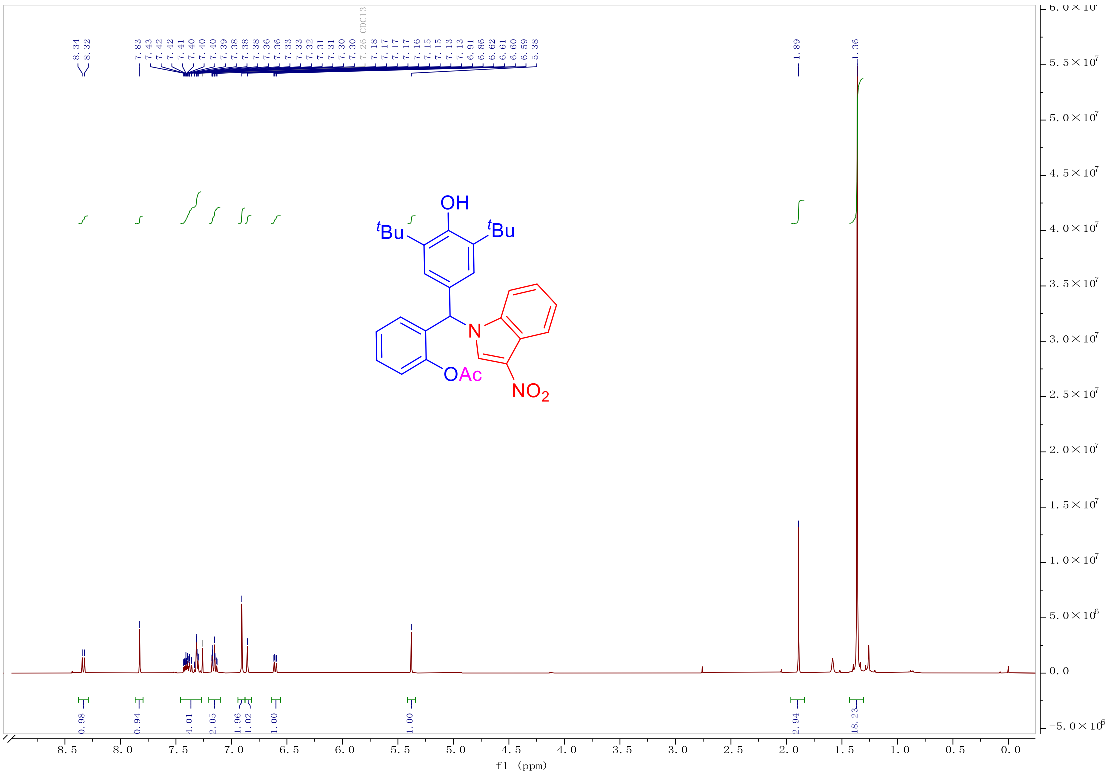

^{13}C NMR spectrum of 3l

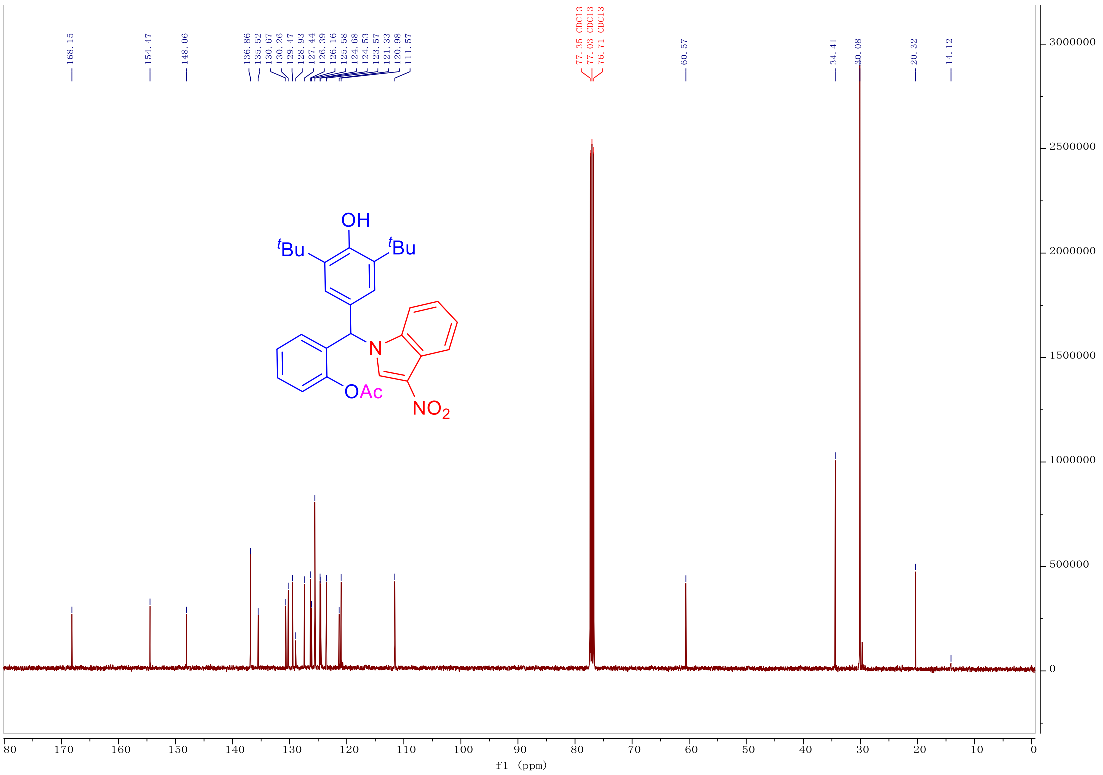

^{1}H NMR spectrum of 3m

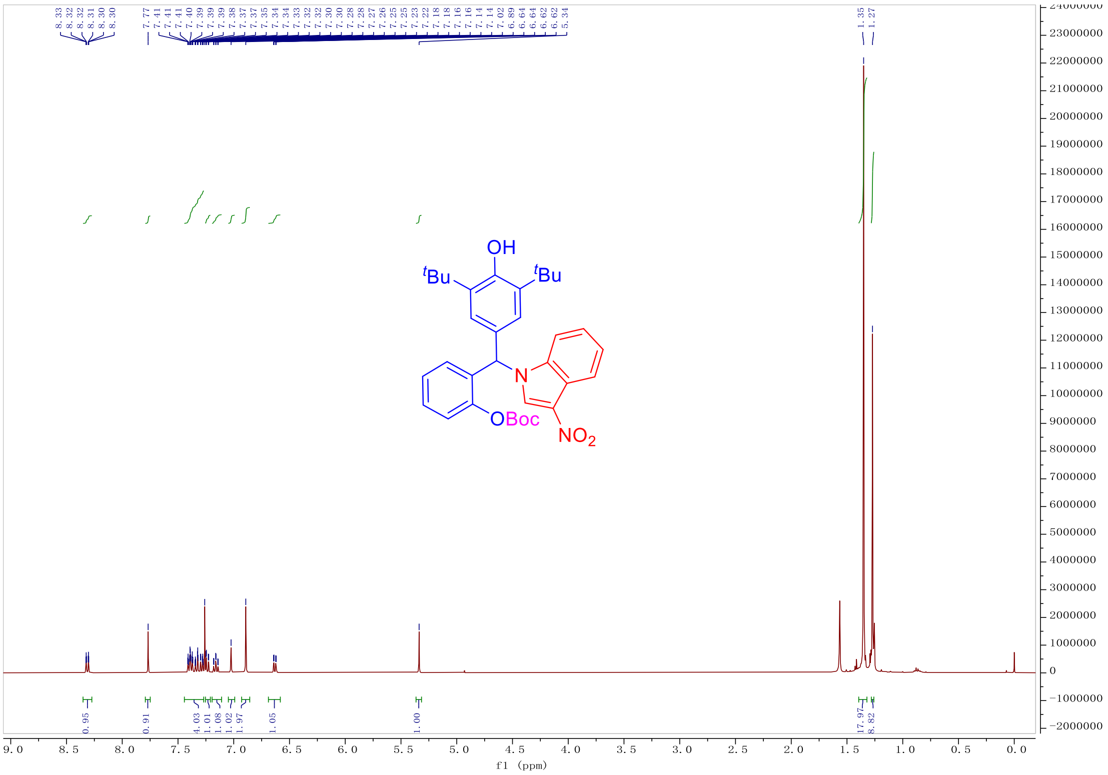

^{13}C NMR spectrum of 3m

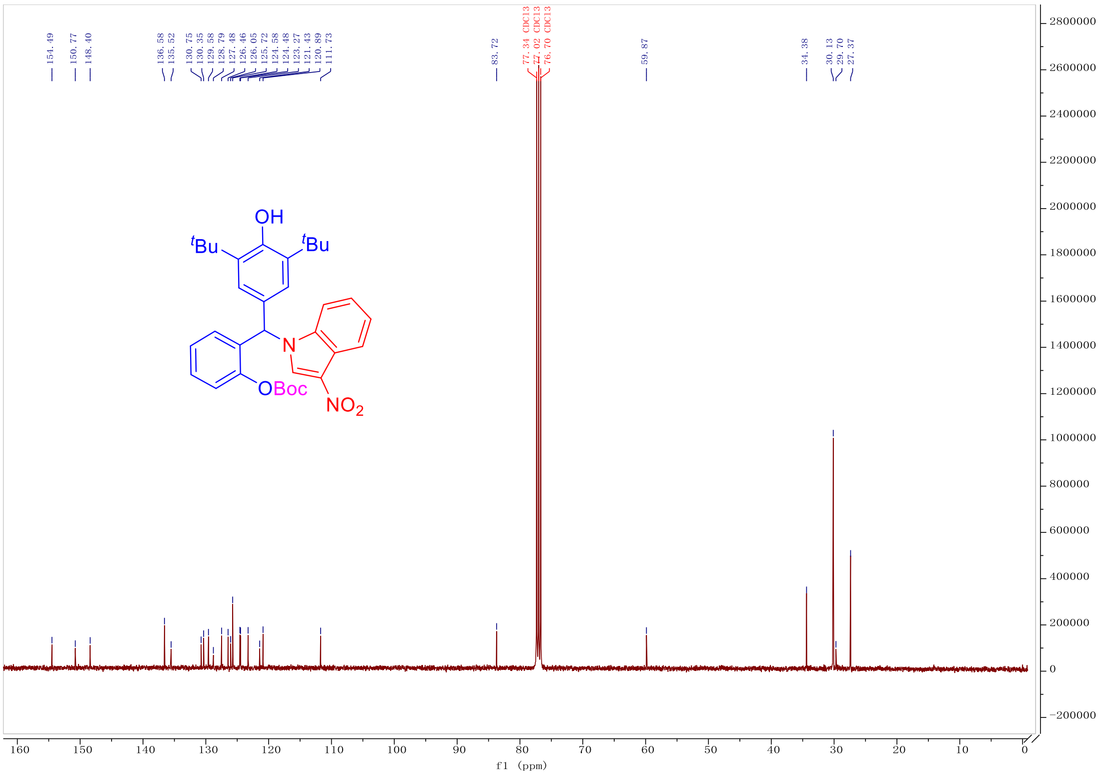

^{1}H NMR spectrum of 3n

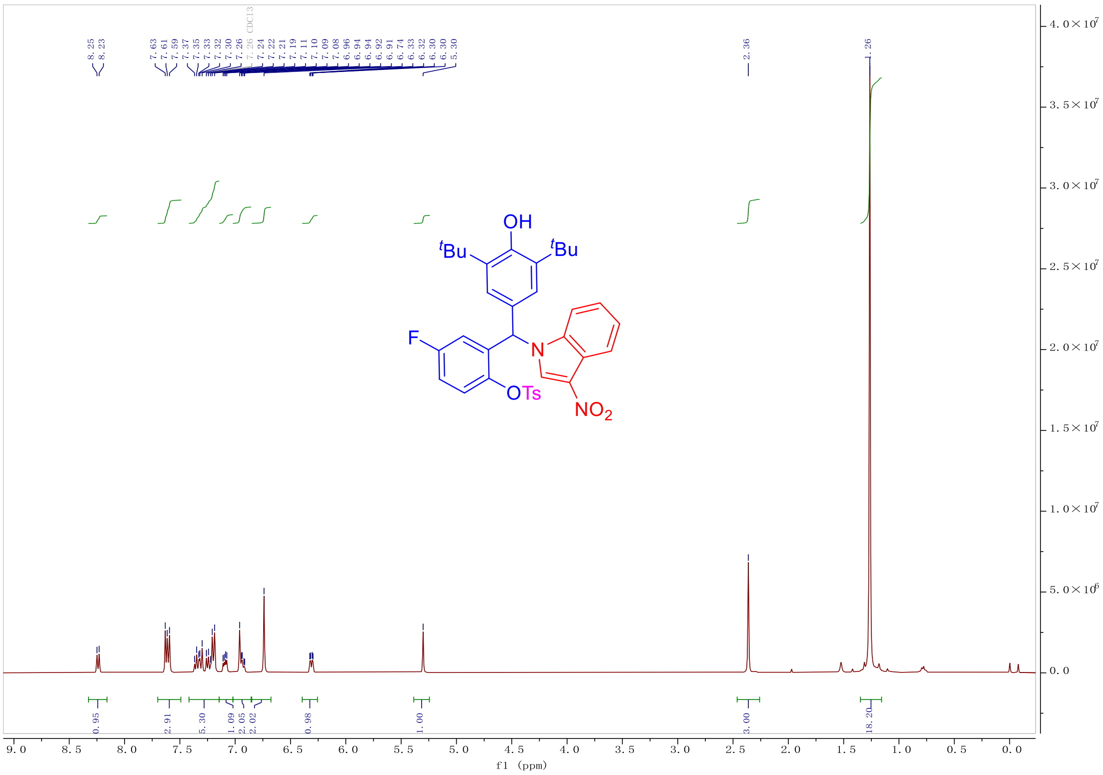

^{13}C NMR spectrum of 3n

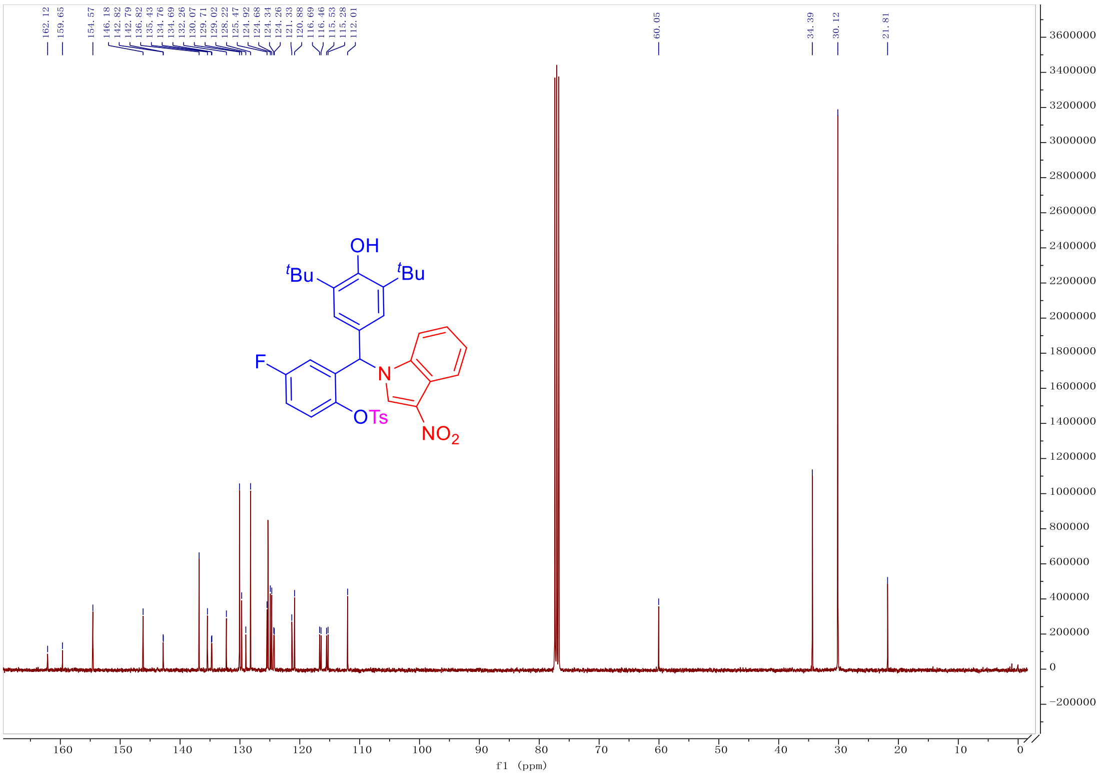

^{1}H NMR spectrum of 3o

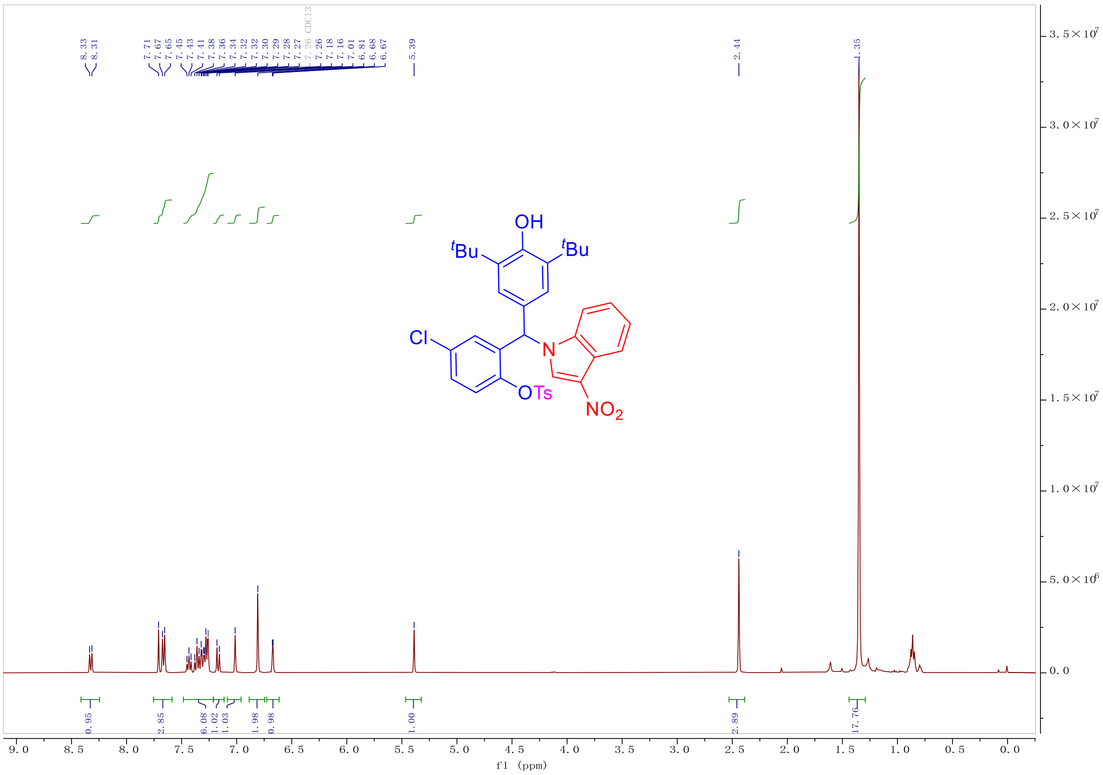

^{13}C NMR spectrum of 3o

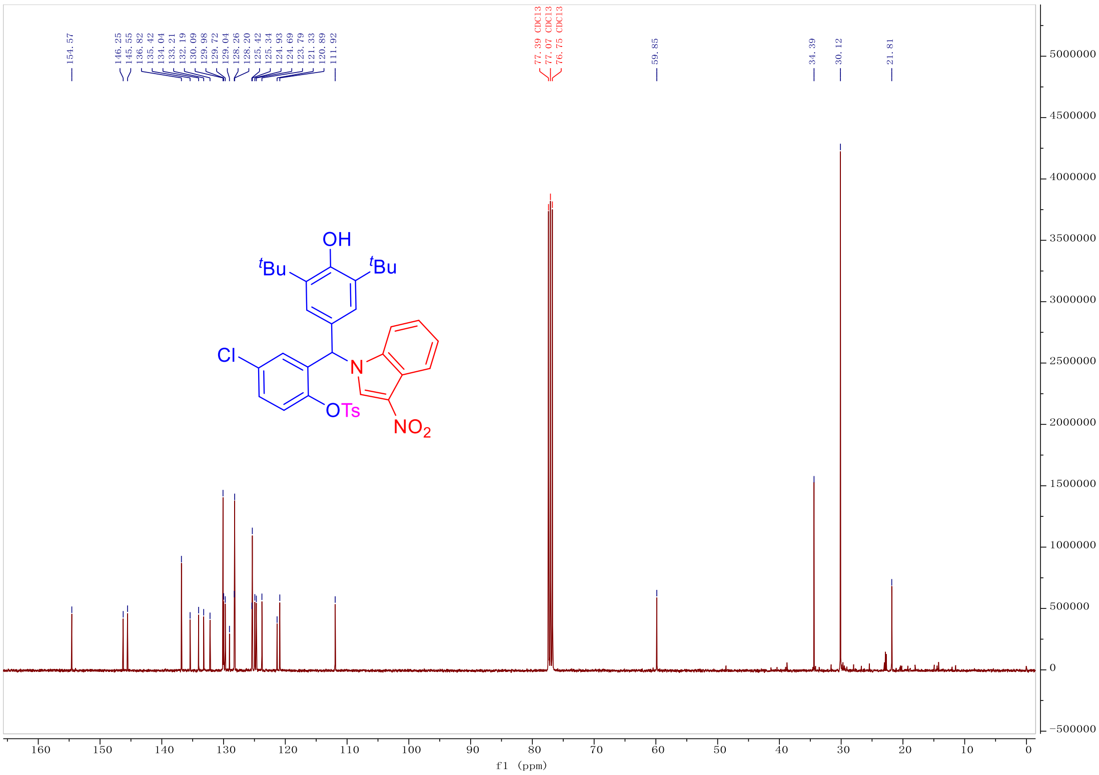

^{1}H NMR spectrum of 3p

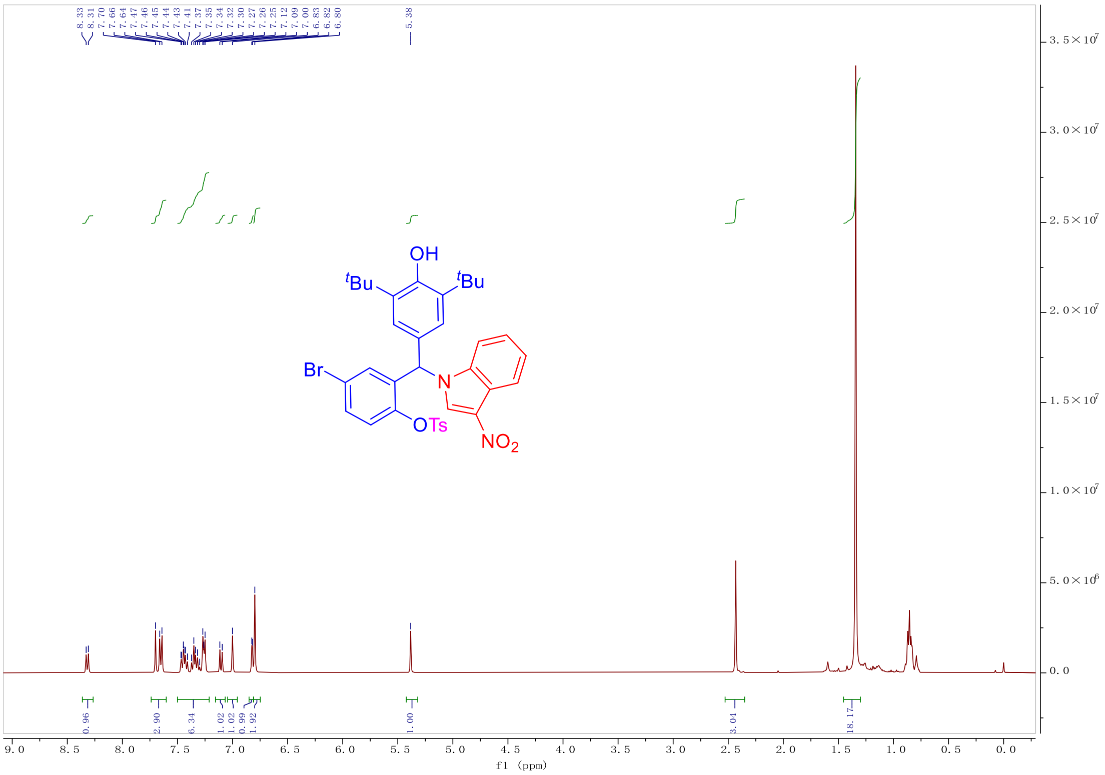

^{13}C NMR spectrum of 3p

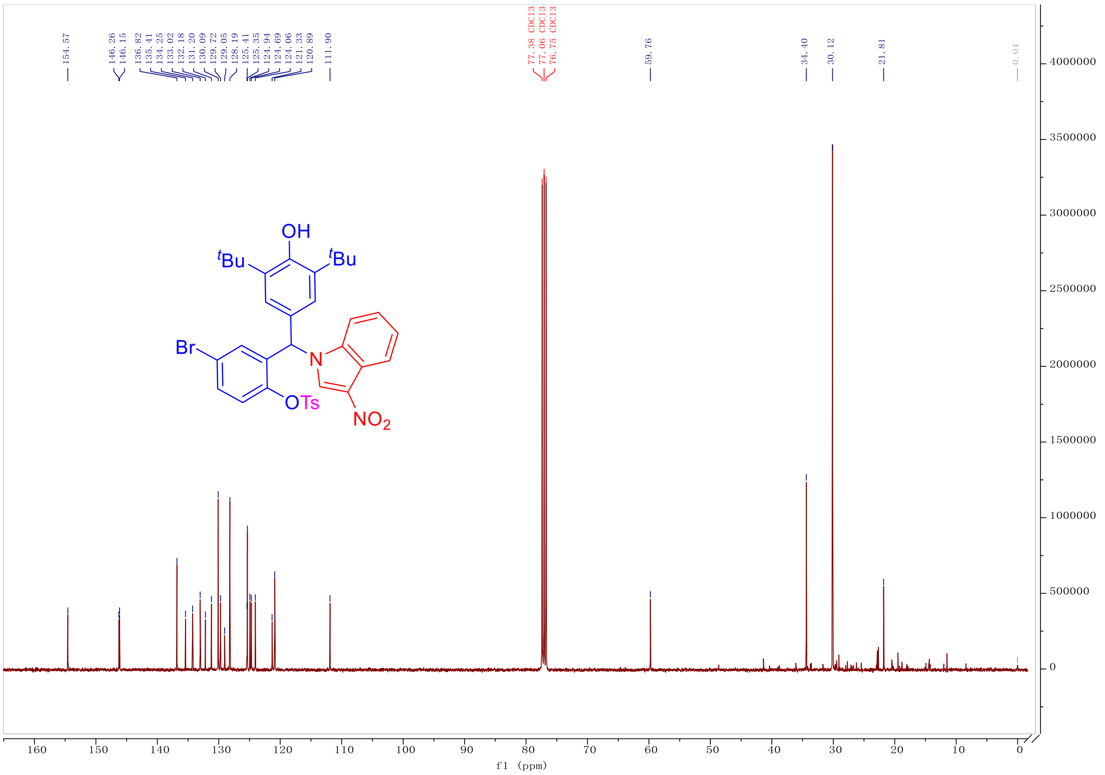

^{1}H NMR spectrum of 3q

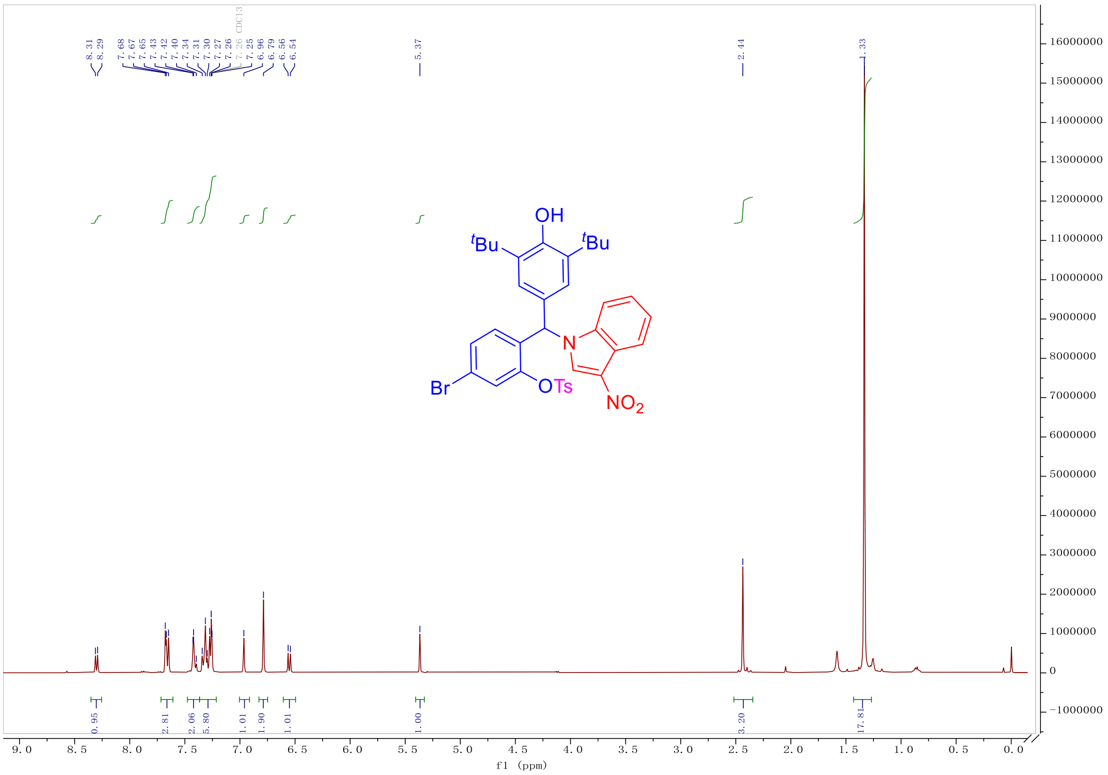

^{13}C NMR spectrum of 3q

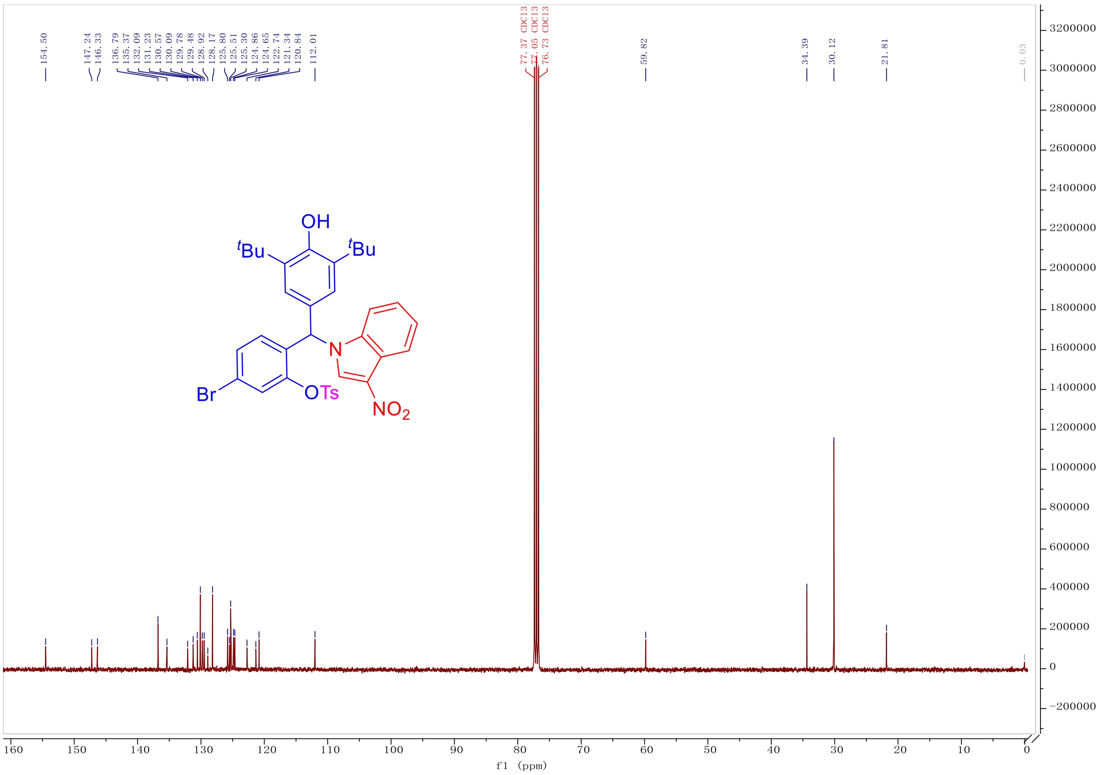

^{1}H NMR spectrum of 3r

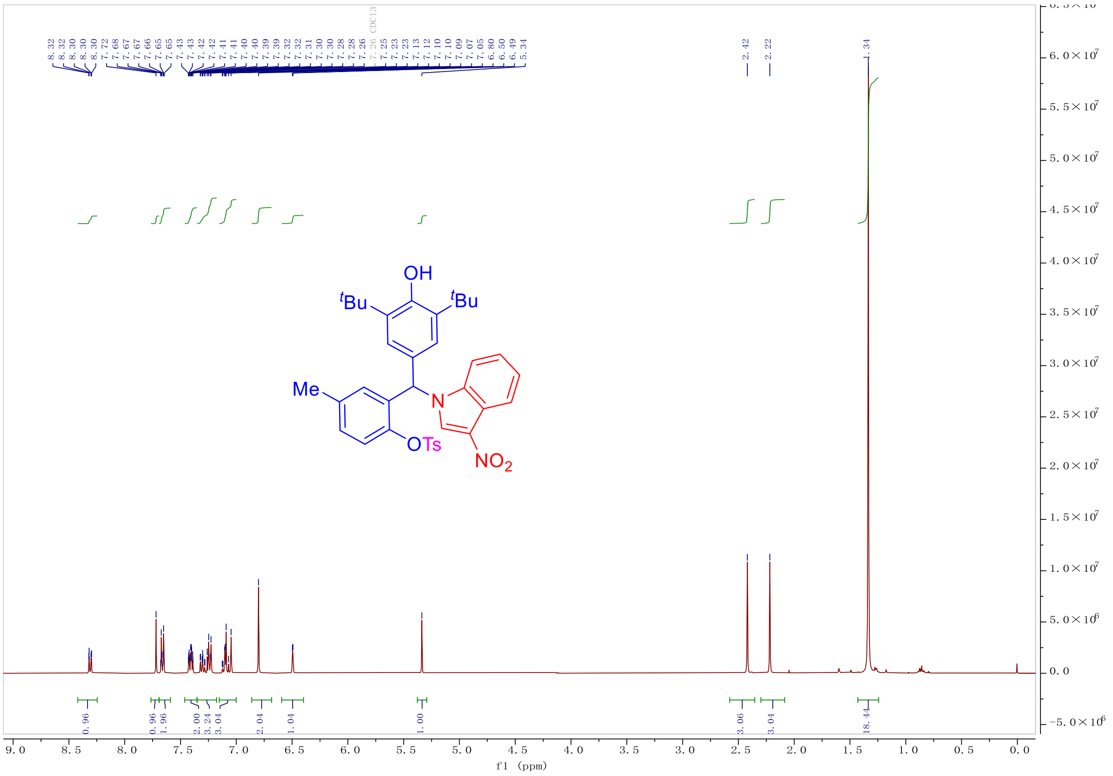

^{13}C NMR spectrum of 3r

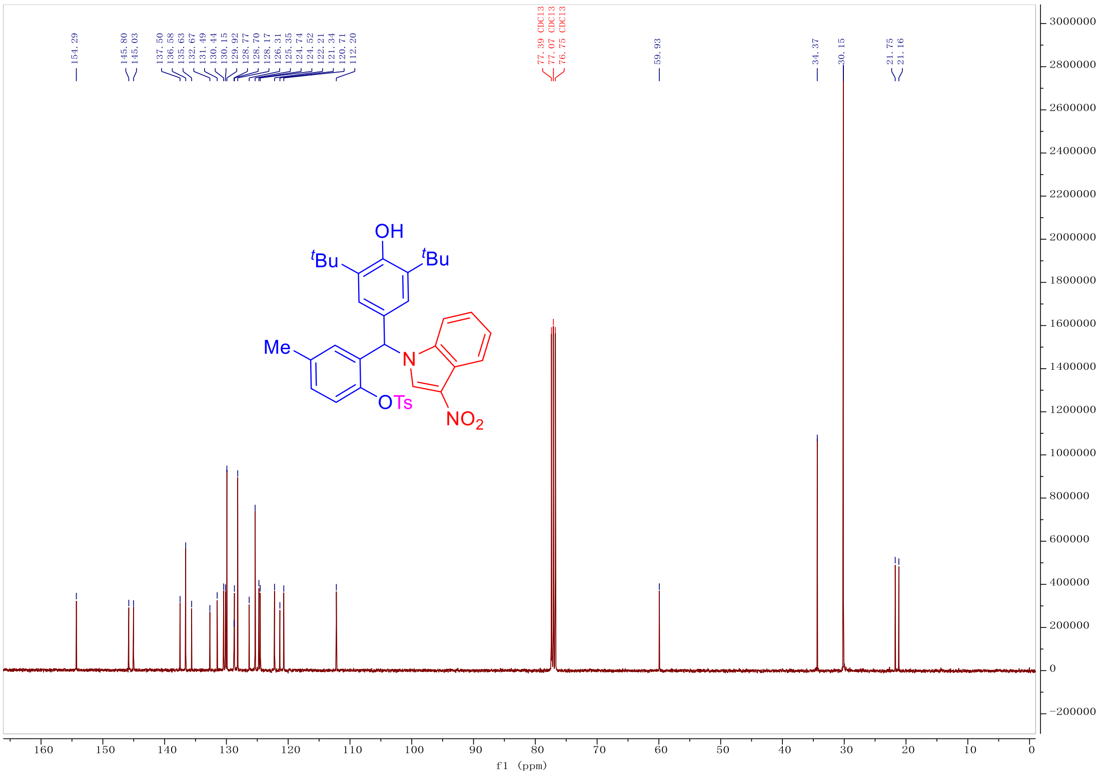

^{1}H NMR spectrum of 3s

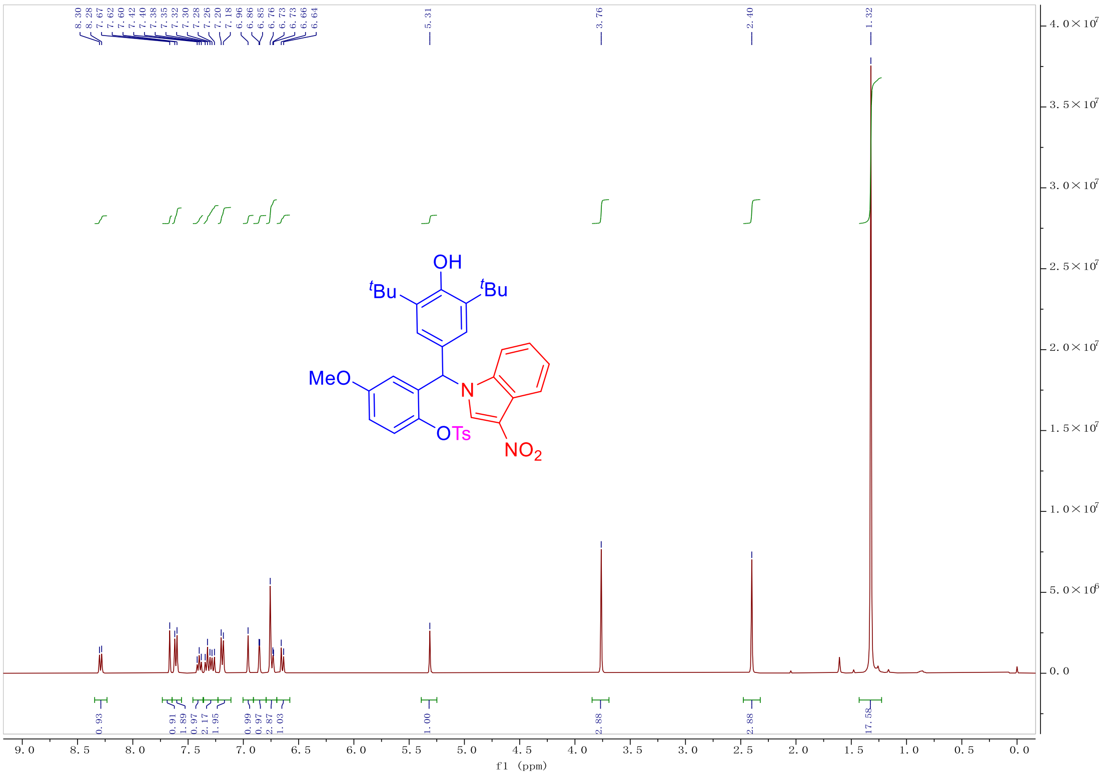

^{13}C NMR spectrum of 3s

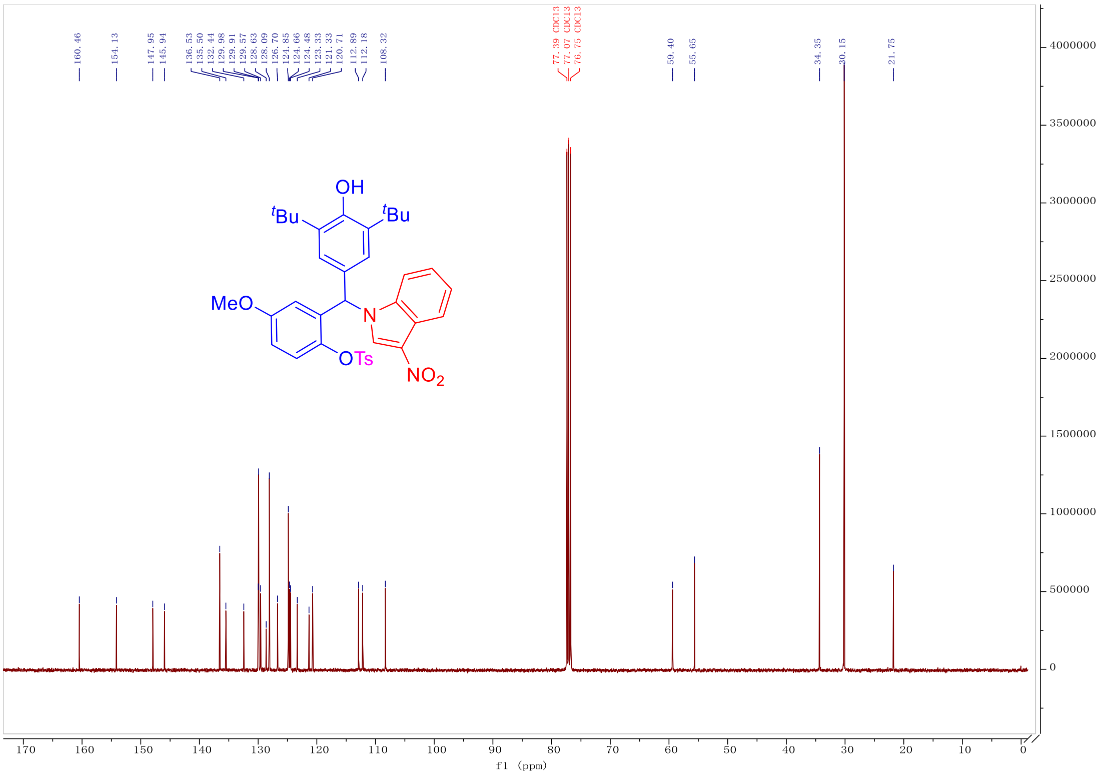

^1H NMR spectrum of 3t

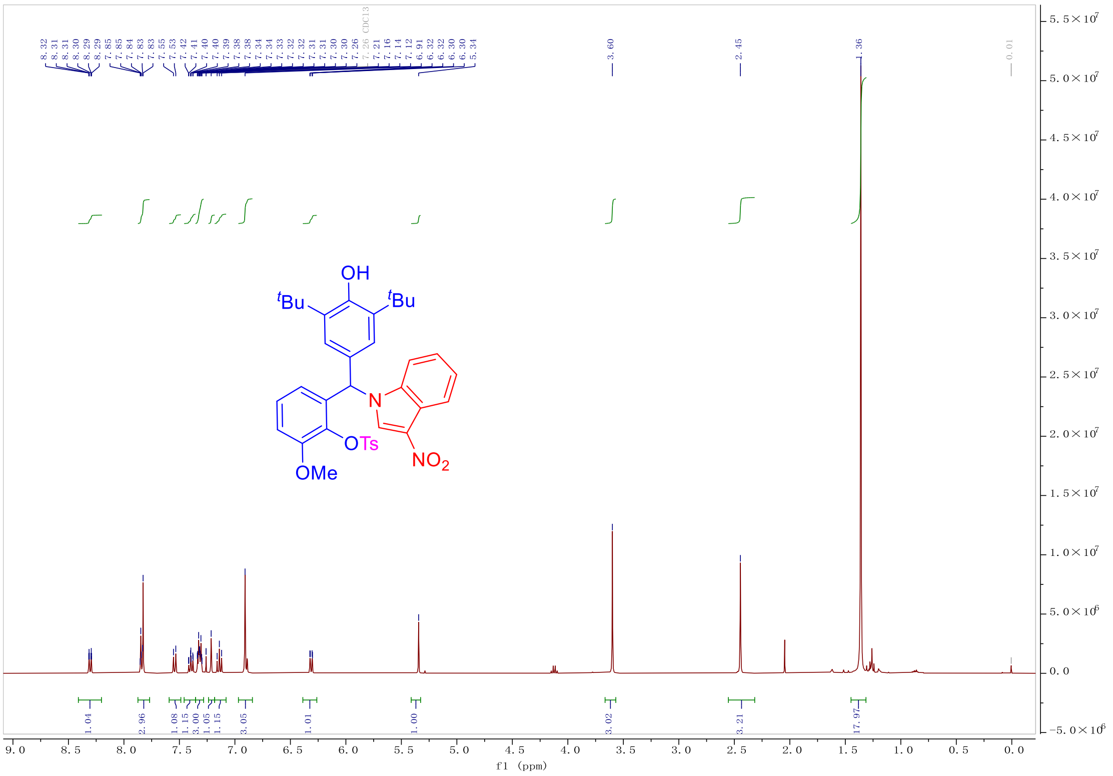

^{13}C NMR spectrum of 3t

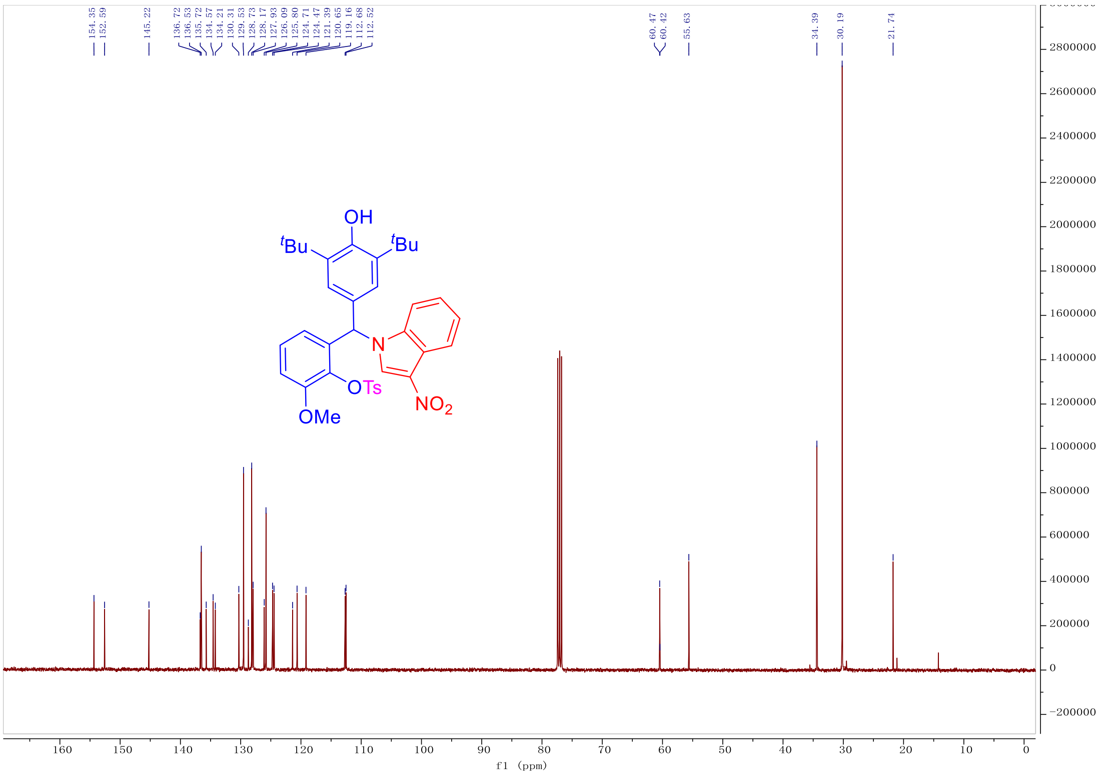

^{1}H NMR spectrum of 6

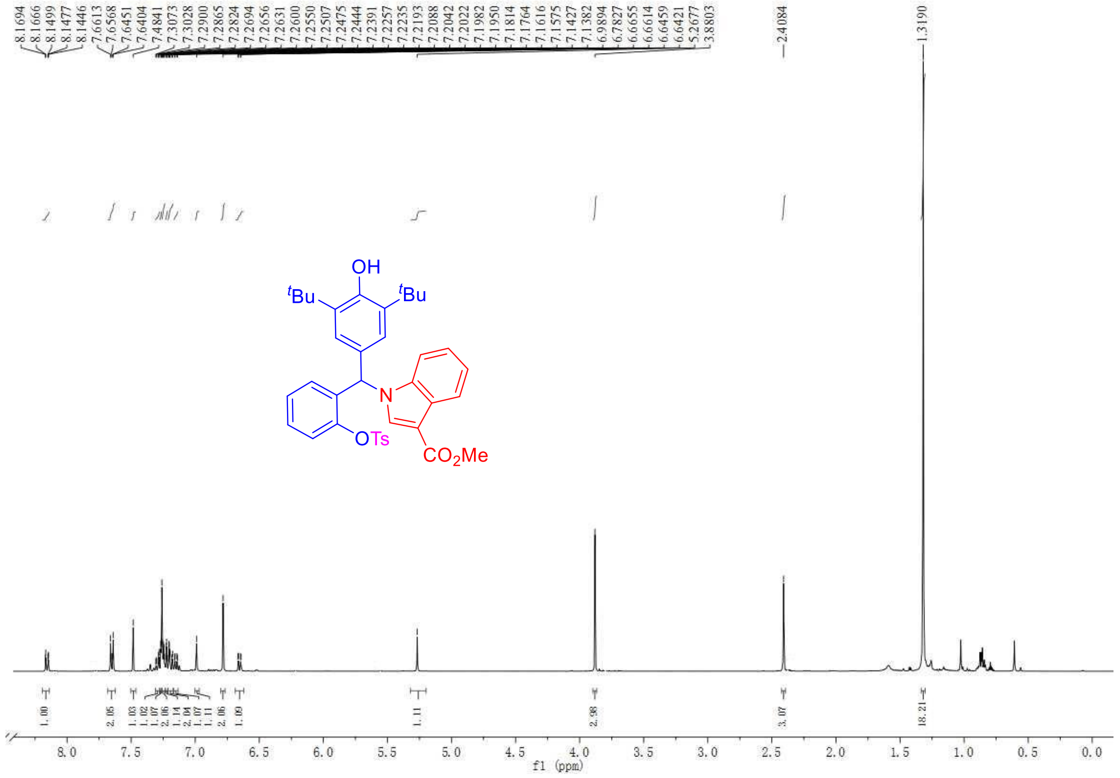

^{13}C NMR spectrum of 6

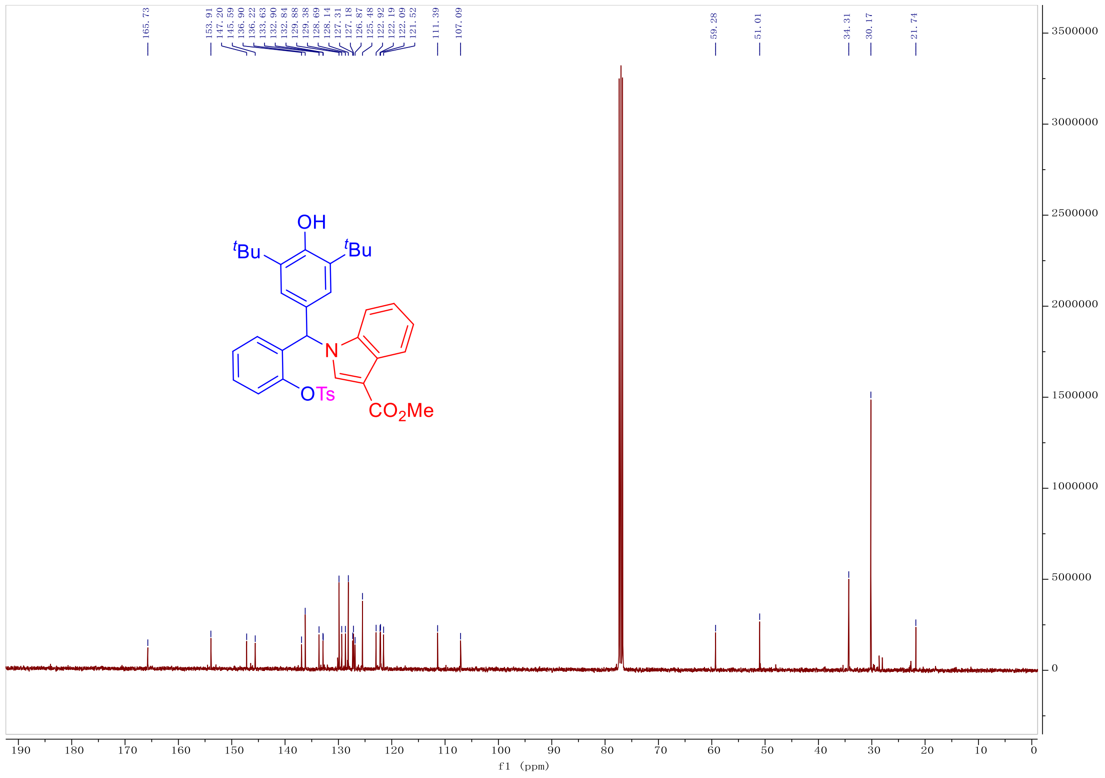

^{1}H NMR spectrum of 11

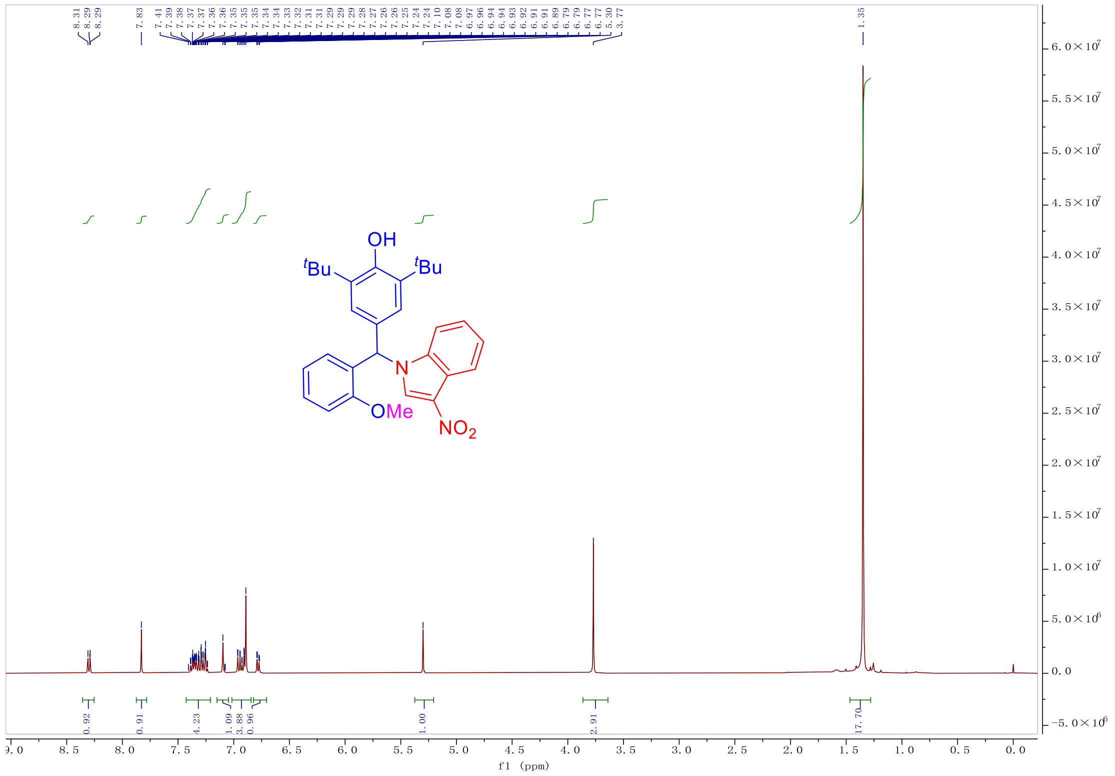

^{13}C NMR spectrum of 11

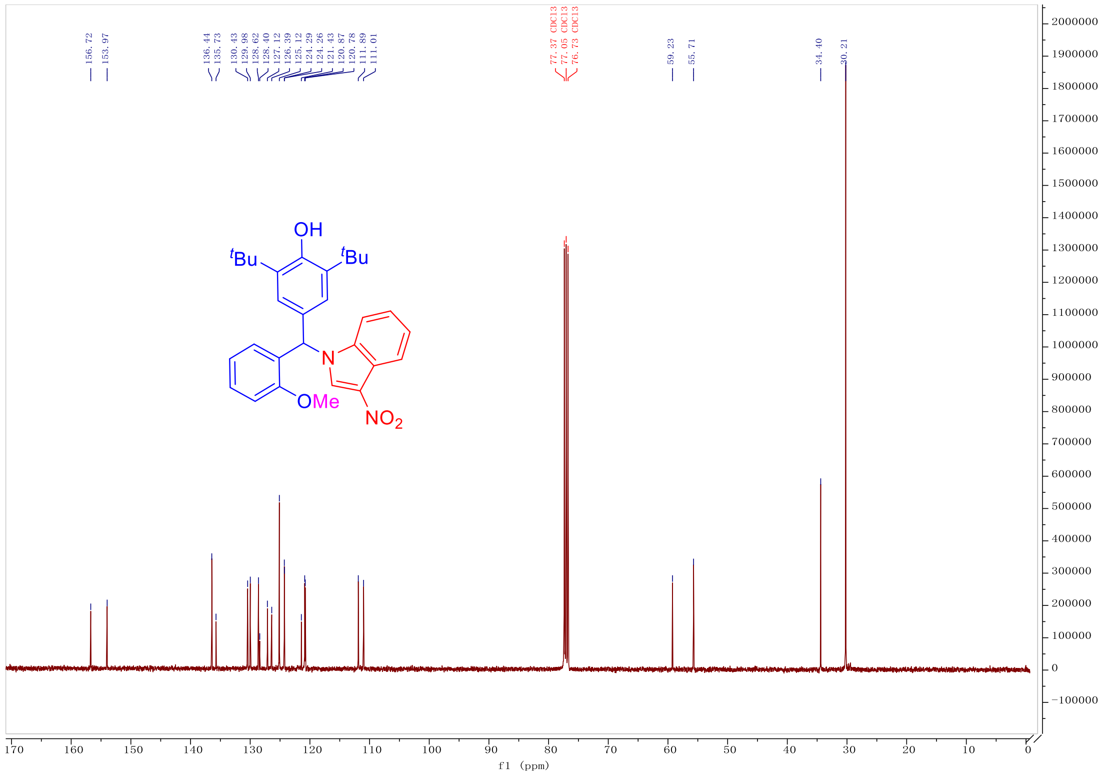

Supplement: Supplementary file 1 [file molecules-28-05529-s001.zip › molecules-2512728-supplementary.pdf]
